# Autophagy of the somatic stalk cells nurses the propagating spores of Dictyostelid social amoebas

**DOI:** 10.12688/openreseurope.14947.1

**Published:** 2022-09-01

**Authors:** Qingyou Du, Pauline Schaap

**Affiliations:** 1 School of Life Sciences, University of Dundee, Dundee, Angus, DD15EH, UK

**Keywords:** evolution of multicellularity, evolution of soma, autophagy, sporulation, encystation, Dictyostelia

## Abstract

**Background:**  Autophagy (self-feeding) assists survival of starving cells by partial self-digestion, while dormancy as cysts, spores or seeds enables long-term survival. Starving *Dictyostelium* amoebas construct multicellular fruiting bodies with spores and stalk cells, with many Dictyostelia still able to encyst individually like their single-celled ancestors. While autophagy mostly occurs in the somatic stalk cells, autophagy gene knock-outs in *Dictyostelium discoideum *( *D. discoideum*) formed no spores and lacked cAMP induction of prespore gene expression.

**Methods:** To investigate whether autophagy also prevents encystation, we knocked-out autophagy genes *atg5* and *atg7* in the dictyostelid *Polysphondylium pallidum*, which forms both spores and cysts. We measured spore and cyst differentiation and viability in the knock-out as well as stalk and spore gene expression and its regulation by cAMP. We tested a hypothesis that spores require materials derived from autophagy in stalk cells. Sporulation requires secreted cAMP acting on receptors and intracellular cAMP acting on PKA. We compared the morphology and viability of spores developed in fruiting bodies with spores induced from single cells by stimulation with cAMP and 8Br-cAMP, a membrane-permeant PKA agonist.

**Results:** Loss of autophagy in *P. pallidum* reduced but did not prevent encystation. However, spore, but not stalk differentiation, and cAMP-induced prespore gene expression were lost. Spores induced *in vitro* by cAMP and 8Br-cAMP were smaller and rounder than spores formed multicellularly and while they were not lysed by detergent they did not germinate, unlike multicellular spores.

**Conclusions:** The stringent requirement of sporulation on both multicellularity and autophagy, which occurs mostly in stalk cells, suggests that stalk cells nurse the spores through autophagy. This highlights autophagy as a major cause for somatic cell evolution in early multicellularity.

## Plain language summary

Autophagy or self-feeding is a process where cells survive starvation by enclosing and digesting part of their contents. Many organisms survive long-term starvation by differentiating into walled dormant cysts or spores. Social amoebas (Dictyostelia) display an early form of multicellularity where starving amoebas aggregate and form a fruiting body where stalk cells support a ball of spores. Like their ancestors, the solitary amoebas, many social amoebas can still form cysts individually. However, cysts have thinner walls than spores and lack frost resistance.

Because starving amoebas require 24 h to differentiate into cysts, spores or stalk cells, it is likely that autophagy is required for these processes. We deleted essential autophagy genes to investigate whether this is the case. Without autophagy, stalks and cysts were still formed, although the latter were less viable. However, no spores were formed at all, and, remarkably, the loss of autophagy already prevented the first step in sporulation, the expression of prespore genes.

Differentiating stalk cells contain many autophagic vesicles and eventually digest their entire contents. However, autophagic vesicles are rare in prespore cells, which mostly contain vesicles where the spore wall is prefabricated. We investigated whether the spores actually benefit from the autophagy in the stalk cells. We compared the properties of spores developed in fruiting bodies, to spores induced in isolation by treatment with spore-inducing stimuli, which are normally produced inside the fruiting bodies. The spores formed in isolation were smaller and non-viable after treatment with detergent, which has no effect on normal spores. These data support the hypothesis that in multicellular structures the autophagy of stalk cells is providing spores with nutrients for proper differentiation. The data more broadly suggest an important role for autophagy in the evolution of somatic (non-propagating) cells, which in animals came to form the largest part of the body.

## Introduction

Macroautophagy (further called autophagy) is a deeply conserved survival strategy in eukaryotes, whereby starving cells gain nutrients by enclosing and digesting cytoplasm and/or organelles. Autophagy also acts in non-starved cells to digest and recycle damaged proteins and organelles, with defective autophagy causing major organ pathologies, metabolic and immune deficiencies and neurodegenerative diseases ( Klionsky *et al.*, 2021). Although long recognized as a cellular function ( Deter *et al.*, 1967), the identification of many conserved autophagy genes through yeast genetics ( Ohsumi, 1999) greatly expanded mechanistic understanding of autophagy and its importance for cellular homeostasis in animals and plants. Briefly, autophagy is initiated by encircling of cytoplasm and/or organelles by a double membrane structure, the isolation body, that closes to form a vesicle, the autophagosome. Fusion with an acidic primary lysosome with digestive enzymes turns the autophagosome into an autolysome and initiates digestion of its contents. The autophagy (Atg) proteins act to sense nutrient status and to initiate and regulate the nucleation of the isolation body at the endoplasmic reticulum and its further expansion, closure and fusion with primary lysosomes ( Nakatogawa, 2020). The majority of *atg* genes are also present in protists, such as the social amoeba *Dictyostelium discoideum* ( *Ddis*) and its solitary ancestors. The experimental and genetic accessibility of *Ddis* has been useful to reveal novel genes and mechanistic insights into autophagy ( Tornero-Écija *et al.*, 2022; Xiong *et al.*, 2018).

We investigate the evolution of multicellularity and cell-type specialization in dictyostelid social amoebas. These amoebas aggregate when starved to form multicellular fruiting bodies. In three out of four of the major dictyostelid taxon groups these structures consist of two cell types, dead stalk cells and dormant spores. However, group four species evolved two more cell types that form a basal disc to support the stalk, and an upper and lower cup to raise and bracket the spore mass ( Schilde *et al.*, 2014). Group four fruiting bodies are also larger than those in groups one to three and group four spores combine relatively large size with a thicker spore wall and higher state of dehydration ( Lawal *et al.*, 2020; Romeralo *et al.*, 2013). These features are correlated with greater frost resistance and colonization of arctic and alpine habitats by group four species ( Lawal *et al.*, 2020).

Both spore and stalk cell maturation require activation of cAMP-dependent protein kinase (PKA) by cAMP ( Harwood *et al.*, 1992; Hopper *et al.*, 1993), while spore differentiation additionally requires extracellular cAMP acting on surface cAMP receptors (cARs) ( Schaap & Van Driel, 1985). Comparative studies across Dictyostelia and the ancestral solitary Amoebozoa showed that the roles of intracellular cAMP and PKA on spore and stalk maturation are evolutionary derived from roles as intermediates for starvation and drought-induced encystment in solitary amoebas ( Kawabe *et al.*, 2015; Ritchie *et al.*, 2008). cAR mediated (pre) spore gene induction is however restricted to Dictyostelia ( Kawabe *et al.*, 2009). To gain insight into the mechanisms regulating sporulation, we performed a genetic screen for sporulation-deficient mutants in the group four species *Ddis.* This screen yielded a transcription factor, SpaA, as being essential for sporulation ( Yamada *et al.*, 2018), but also the autophagy gene *atg7* ( Yamada & Schaap, 2019) and two novel autophagy genes *knkA* and *bcas3* ( Yamada & Schaap, 2021). While it was expected that lack of autophagy would impact on spore viability, the autophagy-deficient mutants were specifically defective in cAR-mediated induction of prespore gene expression by cAMP. The same defect was displayed by knock-outs (KOs) in *atg5* and *atg9* ( Yamada & Schaap, 2019).

To investigate whether this unexpected involvement of autophagy with cAMP signal transduction is conserved in Dictyostelia and whether loss of autophagy also impacts on encystation, we disrupted the autophagy genes *atg7* and *atg5* in *Polysphondylium pallidum,* a group two species that can both sporulate in multicellular fruiting bodies or encyst individually when starved. Group four species have lost this ancestral survival strategy. The *P. pallidum* strain PN500_J ( *Ppal*) *atg7* and *atg5* KOs still formed cysts but both their number and ability to regrow was reduced. The mutants aggregated and extended short thick stalks, but no spores were formed and cAMP induction of prespore gene expression was lost. In both *Ddis* and *Ppal,* prestalk and stalk cells display much more autophagy than prespore cells. The relatively mild effect of defective autophagy on encystation and stalk cell differentiation led us to test a hypothesis that the spores require nutrients provided by autophagy of the prestalk/stalk population. This hypothesis found support in further experimentation showing that spores induced individually by incubation of cells with spore-inducing signals were almost completely non-viable compared to spores formed (by the same signals) in fruiting bodies. We developed these observations into a new model for dictyostelid evolution, whereby the somatic stalk cells do not only act to lift the spore mass, but to nurture the spores and render them more resilient to environmental stress than the individually developing cysts. This places autophagy at the forefront of ultimate causes for somatic cell evolution.

## Methods

### Cell culture

*P. pallidum* strain PN500_J ( *Ppal*) was grown in association with *Klebsiella aerogenes* at 22°C on 0.1% lactose-peptone (LP) (1 g Lactose (BDH, UK), 1 g Bacto ^TM^ Peptone (Gibco; Thermo Fisher Scientific, Inc.), 2.2 g KH _2_PO _4_ (VWR), 1.25 g Na _2_HPO _4_·2H _2_O (VWR) and 15 g agar in 1 L H _2_O) or 1/5 ^th^ SM (Formedium) agar plates. PN500_J is an isolate of *Ppal* PN500 with more robust multicellular development. For multicellular development, *Ppal* cells were harvested in KK2 (20 mM K-phosphate, pH 6.2) and distributed on non-nutrient (NN) agar (1.5% agar in 8.8 mM KH _2_PO _4_ and 2.7 mM Na _2_HPO _4_) at 10 ^6^ cells/cm ^2 ^and incubated at 22°C.

### Plasmid constructs

**Figure 1.**
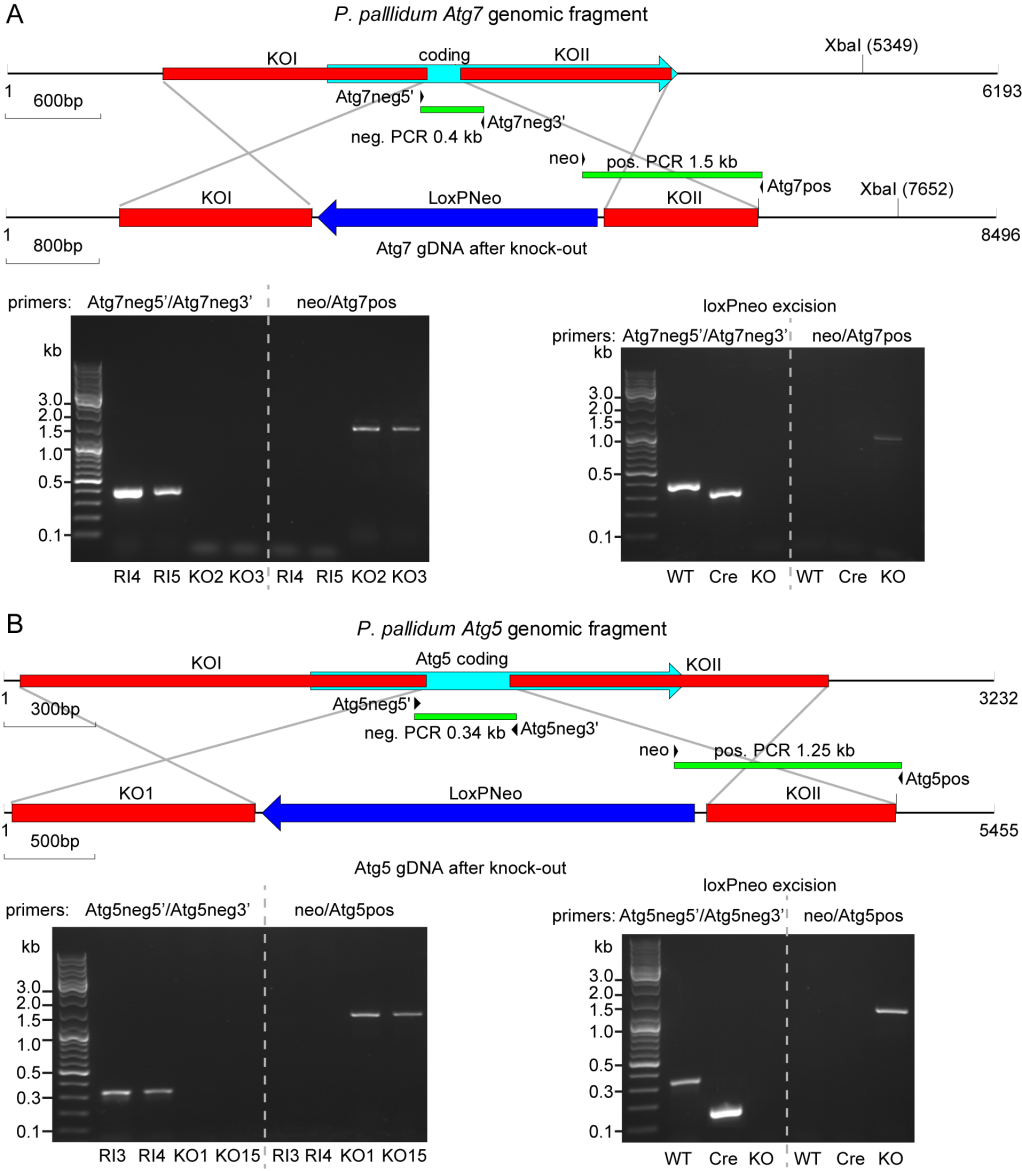
Disruption of the *P. pallidum* atg7 and atg5 genes. ***A***. *Atg7*. Top: schematic of the *P. pallidum* strain PN500_J ( *Ppal*) *Atg7* genomic region with the positions of the two fragments knock-out (KO)I and KOII before and after homologous recombination with the LoxPNeo KO construct. Primer pair Atg7neg5’/Atg7neg3’ amplifies a 0.4 kb fragment in wild-type (WT) and random integrant (RI) only, while primer pair neo/Atg7pos amplifies a 1.5 kb fragment in *atg7* KOs only. Bottom left: Diagnostic PCRs of two RI and two KO clones. Bottom right: Diagnostic PCR of a KO clone after excision of LoxPNeo with Cre-recombinase (Cre) showing the expected 220 bp product after amplification with primer pair Atg7neg5’/Atg7neg3’, and absence of product with primer pair neo/Atg7pos. ***B***. *Atg5*. Top: schematic of the *Ppal Atg5* genomic region with the positions of the two fragments KOI and KOII before and after homologous recombination with the LoxPNeo KO construct. Primer pair Atg5neg5’/Atg5neg3’ amplifies a 0.34 kb fragment in WT and RI, while primer pair neo/Atg5pos amplifies a 1.25 kb fragment in *atg5* KOs. Bottom left: Diagnostic PCRs of two RI and two KO clones. Bottom right: Diagnostic PCR of a KO clone after excision of LoxPNeo with Cre-recombinase (Cre) showing the expected 180 bp product after amplification with primer pair Atg5neg5’/Atg5neg3’, and absence of product with primer pair neo/Atg7pos.

***atg7 and atg5 gene disruption***. To disrupt *Ppal atg7* (PPL_02507), two fragments, I and II, were amplified from *Ppal* genomic DNA by PCR using Phusion DNA polymerase (Thermo Fisher Scientific, Inc.) and primer pairs Atg7-I5’/Atg7-I3’ and Atg7-II5’/Atg7-II3’ ( Table 1), respectively. These primers were based on the *atg7* sequence of *Ppal* PN500_J (GenBank accession: ON758339), since the PN500 *atg7* sequence archived in GenBank ( Heidel *et al.*, 2011) is of poor quality. Fragments I and II were digested with *Sac*I/ *Xba*I or *Xho*I/ *Kpn*I, respectively, using restriction sites included in the primer design, and sequentially inserted using T4 ligase into *Sac*I/ *Xba*I and *Xho*I/ *Kpn*I digested plasmid pLox-NeoIII ( Kawabe *et al.*, 2012) to flank the LoxP-neo selection cassette ( Figure 1A). pLox-NeoIII contains both the *AmpR* gene for selection on ampicillin in *Escherichia coli* XL1-Blue (Agilent) and the NeoR gene for selection on G418 in *Ppal*.

**Table 1.** Oligonucleotide primers used in this work.

Name	Sequence	Restriction site
Atg7-I5’	GATgagctcAAACAGGAAAAGAGGAG	*Sac*I
Atg7-I3’	GATtctagaACGATCCTAACGGTTGAC	*Xba*I
Atg7-II5’	GATctcgagCAATCCTGGTTGGCCACT	*Xho*I
Atg7-II3’	GATggtaccCAATCGATTGATACACCT	*Kpn*I
Atg5-I5’	GATtctagaCGCATACCTATCACTCTTTC	*Xba*I
Atg5-I3’	GATggatccAGCATGTCAAAGAGTACA	*Bam*HI
Atg5-II5’	GATgtcgacCAAACCAATGAGTTATGGG	*Sal*I
Atg5-II3’	GATggtaccTTTGGGATATATCGGA	*Kpn*I
Atg7neg5’	ACTCCTATCACTACGGTTGG	
Atg7neg3’	CTCAGTTGGGAGGTGTACAG	
Atg7pos	CATCGTCAGACGATGATTCT	
Atg5neg5’	CAAGTGGCACATTCCAATCG	
Atg5neg3’	ACCATCCCATAACTCATTGG	
Atg5pos	GTACATTGAGACCAGCGGTG	
Neo	GGGCAAATCTGTAATTTTCAG	
Atg7-g5’	GATgaattcATGTCAAATAATGAAGAGATTT	*Eco*RI
Atg7-g3’	GATctcgagTTATTCATCGTCAGACGATG	*Xho*I
Atg7-pro5’	GATgctagcTTGGTGTTGTTGATCAGG	*Nhe*I
Atg7-pro3’	GATggatccAAAATCTCTTCATTATTTGACAT	*Bam*HI
Atg5-g5’	GATggatccATGTCATTCTTTGATGAAGATG	*Bam*HI
Atg5-g3’	GATactagtCATGCCAATACAATATATAAA	*Spe*I
Atg5-pro5’	GATgctagcGATGATATGATGTCTGAATG	*Nhe*I
Atg5-pro3’	GATggatccTTTTATGGATATGATATACGC	*Bam*HI
SP45P1	GATGGTCAACAACGTTGCCA	
SP45P2r	TTGGCGATGGGAACTGGTGC	
PPL_04427 P1	CTGTACCTACGACAGCTGCT	
PPL_04427 P2r	TGTTGTCCTTGCAGTAGTCG	
PPL_07209 P1	TGGCTTGGATCAACACTCCA	
PPL_07209 P2r	ATGAACCACGGATGGTGTGA	

Restriction enzymes sequences used for cloning are shown in lower case.

To disrupt *Ppal atg5* (PPL_04841), DNA fragments were amplified similarly as described above, using primer pairs Atg5-I5’/Atg5-I3’ and Atg5-II5’/Atg5-II3’, which contained *Xba*I/ *Bam*HI and *Sal*I/ *Kpn*I restriction sites, respectively ( Table 1), and inserted in pLox-NeoIII ( Figure 1B). Restriction enzymes and T4 ligase were purchased from New England Biolabs (NEB). All reactions were performed with recommended buffers according to the manufacturer's instructions. All DNA constructs were validated by sequencing.

For transformation, the KO fragments were excised with *Sac*I/ *Kpn*I and *Xba*I/ *Kpn*I for the *atg7* and *atg5* KO plasmids, respectively. *Ppal* cells were harvested from growth plates and starved for 5 h in HL5 (Formedium, UK) at 2.5 × 10 ^6^ cells/ml, followed by resuspension in ice-cold H-50 buffer (4.8 g HEPES (Formedium), 3.7 g KCl, 0.58 g NaCl, 0.25 g MgSO _4_, 0.42 g NaHCO _3_, 0.14 g NaH _2_PO _4 _in 1 L H _2_O). Cells (2.5 × 10 ^6^) were combined with 5 µg KO fragment and 2 nmol flanking primers in a total volume of 100 µl in 1 mm gap cuvettes (BTX) and transformed by electroporation with two pulses of 0.65 kV/25 µFd, separated by a 5 second interval, using a GenPulser2 (BioRad). Recovery and selection of transformants at 300 µg/ml G418 (Formedium) was performed as described before ( Kawabe *et al.*, 1999). Genomic DNAs were isolated from G418 resistant clones using the GenElute™ Mammalian Genomic DNA Miniprep Kit (Sigma) and screened for homologous recombination events by two PCR reactions, using primer pairs Atg7neg5’/Atg7neg3’ neo/Atg7pos ( Table 1) for *atg7* KO diagnosis and primer pairs Atg5neg5’/Atg5neg3’ and neo/Atg5pos ( Table 1) for *atg5* KO diagnosis ( Figure 1).

***Deletion of the Neo resistance cassette***. To remove G418 resistance from *atg7ˉ* and *atg5ˉ*, cells were electroporated with plasmid pDM1483, which contains cre-recombinase ( Paschke *et al.*, 2018) and a Nourseothricin resistance gene, transferred to a petri dish containing autoclaved *K. aerogenes* in KK2 for 24 h and exposed to 300 µg/ml Nourseothricin (Jena Bioscience) for 2-4 days. Resistant clones were replica-plated onto LP agar with autoclaved *K. aerogenes* with and without 300 µg/ml G418 for negative selection of G418 sensitive clones. Loss of the Neo cassette was confirmed by two PCR reactions ( Figure 1).

***Expression constructs***. To express *atg7* from its own promoter, a 1.5 kb region upstream of the start codon was amplified from *Ppal* gDNA using primers Atg7pro5’/Atg7pro3’ ( Table 1), which contain *Nhe*I and *Bam*HI sites, respectively, and inserted into *Nhe*I/ *Bam*HI digested vector pExp5 ( Meima *et al.*, 2007). Next the 2.1 kb *atg7* coding region was amplified from cDNA with primer pairs Atg7-g5’ and Atg7-g3’ ( Table 1) that contain *Eco*RI and *Xho*I sites, respectively, and after digestion joined with the promoter fragment in the *Eco*RI/ *Xho*I digested plasmid, creating pPpalAtg7pAtg7.

To express *atg5* from its own promoter, the 963 bp *atg5* coding region was amplified from *Ppal* cDNA using primer pair Atg5-g5’/Atg5-g3’ with *Bam*HI and *Spe*I sites, respectively, and inserted into *Bam*HI/ *Spe*I digested vector pExp5(+) (GenBank: EF028664.1). Next the 620 bp 5’intergenic region was amplified from gDNA with primer pair Atg5-pro5’/Atg5-pro3’ with *Nhe*I and *Bam*HI sites, respectively, and inserted into the *Nhe*I/ *Bam*HI sites of the same vector, creating pPpalAtg5pAtg5. pPpalAtg7pAtg7 and pPpalAtg5pAtg5 were electroporated into *atg7* ^- ^and *atg5* ^-^ cells, respectively, from which the loxPNeo cassette had been removed with cre-recombinase. Transformants were selected at 300 µg/ml G418.

The amplified *atg7* and *atg5* promoter fragments were also inserted into *Xba*I/ *Bam*HI digested vector pDdGal17 ( Harwood & Drury, 1990), which places the promoters upstream of the *LacZ* reporter gene. The resulting pPpalAtg7-LacZ and pPpalAtg5-LacZ plasmids were transformed into wild-type (WT) *Ppal* and transformed cells were selected at 300 µg/ml G418.

***β-galactosidase histochemistry***. *Ppal* cells, transformed with promoter- *lacZ* constructs, were plated on nitrocellulose filters supported by NN agar at 10 ^6^ cells/cm ^2^ and incubated at 22°C until the desired developmental stages had been reached. Filters with developing structures were transferred to Whatman 3MM chromatography paper, soaked in 0.5% glutaraldehyde, and incubated in a sealed chamber for 6 min. Structures were next fully submersed in 0.5% glutaraldehyde for 3 min. After washing with Z-buffer (10 mM KCl, 1 mM MgSO _4_, 60 mM Na _2_HPO _4_ and 40 mM NaH _2_PO _4_, pH 7.0), structures were stained with X-gal staining solution (5 mM K _3_[Fe(CN) _6_], 5 mM K _4_[Fe(CN) _6_], 1 mM 5-bromo-4-chloro-3-indolyl β-D-galactopyranoside (X-gal) and 1 mM EGTA in Z buffer) (all reagents were from Sigma) as described previously ( Dingermann *et al.*, 1989). Staining times varied between genes, but different developmental stages of cells transformed with the same construct were stained for the same period.

### Chimeric development

*Ppal* WT and *atg5ˉ* or *atg7ˉ* cells were harvested from growth plates and resuspended in KK2. WT and mutant cells were mixed to contain 5% or 20% WT cells and developed for 2 days on NN agar. Structures were imaged and then harvested, shaken for 5 min with 0.1% Triton X-100 in KK2 to lyse unwalled amoebas, and plated with autoclaved *K. aerogenes* on LP agar supplemented with or without 300 µg/ml G418.

### Encystation and cyst germination

To induce encystation, *Ppal* WT and mutant cells were harvested from growth plates, resuspended in encystation medium (400 mM sorbitol in KK2) at 5x10 ^6^ cells/ml and incubated for 3 days until WT cells had formed mature cysts. Mature cysts were visualized by addition of Calcofluor (Sigma) to 0.001%. To induce cyst germination, mature cysts (2–3 days old) were harvested, shaken with 0.1% Triton X-100 and washed with KK2. Cells were counted in a hemacytometer and plated on 1/5 ^th^ SM plates (14 cm Ø) with *K. aerogenes* at 500 cells/plate. The number of emerging plaques was counted after 4 days of incubation at 22°C.

### Developmental and induced gene expression

To measure prespore and prestalk gene expression in early and late sorogens, WT and mutant *Ppal* cells were developed on NN agar for 10 h and 16 h at 22°C. Structures were gently dissociated and harvested for RNA isolation. To measure induction of prespore gene expression by cAMP, *Ppal* cells were developed for 4, 5 or 6 h on NN agar to reach a stage where cells were competent for induction but had not yet started to express prespore genes. After dissociation of aggregates by passage through a 21-gauge needle, cells were resuspended to 5×10 ^6^ cells/ml in 1 mM MgCl _2_ in KK2 and incubated for 4 h in the presence and absence of 1 mM cAMP. Total RNA was isolated from 10 ^7^ cells using the RNAeasy mini kit (Qiagen), DNA contamination was removed using the Turbo DNA-free Kit (Ambion), the RNA concentration was determined using a Multiskan SkyHigh spectrophotometer (Thermo Fisher Scientific, Inc.) and 2 μg RNA was transcribed into cDNA with the sensiFAST cDNA synthesis kit (Bioline), according to the manufacturer's instructions. Using 60 ng of cDNA as template, transcript levels of the prespore gene *sp45* ( *PPL_06034),* the prestalk gene *PPL_04427* and the constitutively expressed gene *PPL_07209* were assessed by reverse transcription-quantitative PCR (RT-qPCR) on a LightCycler® 96 real-time PCR system (Roche) using PerfeCTa SYBR Green SuperMix (Quanta biosciences, USA) and the primers listed in Table 1. Data were normalized to quantification cycle (Cq) values of control samples ( Livak & Schmittgen, 2001) as indicated in the figure legends.

### Staining with anti-spore antibodies

Cells were allowed to attach to 8-well slide glass wells and fixed in ice-cold 85% methanol. After washing with 5% bovine serum albumen (BSA) in PBS, cells were incubated for 16 h at 4°C with 1:5000 diluted custom-made polyclonal antibody (Cambridge Research Biochemicals) raised in rabbit against a 1:1 mixture of *Ppal* and *Ddis* spores that had been pre-adsorbed to an equal volume pellet of methanol-fixed *Ppal* and *Ddis* vegetative cells ( Schilde *et al.*, 2014). After washing, cells were incubated with 1:2000 diluted polyclonal Alexa fluor 488-conjugated goat anti-rabbit IgG (Thermo Fisher Scientific Cat# A32731, RRID:AB_2633280) for 4 h at 21°C and imaged using a DMLB2 fluorescence microscope (Leica) and Micropublisher 3.3 camera (Qimaging).

### *In vitro* spore induction and germination

WT *Ddis* Ax2 or Ax2/cotC-mRFP cells were harvested from growth plates and resuspended at 10 ^6^ cells/ml in spore salts (20 mM KCl, 20 mM NaCl, 1 mM CaCl _2_ and 1 mM MgCl _2_), supplemented with 5 mM cAMP. Cells were incubated as 1 ml aliquots in 6-well plates (~10 ^5^ cells/cm ^2^) at 22°C without further additives (control) or with 15 mM 8Br-cAMP (8-bromoadenosine 3’:5’-monophosphate; Biolog, Germany). After 30 h, the cells were stained with 0.001% Calcofluor, photographed under phase contrast and epifluorescence illumination.

The cross-section area and length and width of Calcofluor positive spores were quantitated using ImageJ v1.53e (RRID:SCR_003070).

To test germination and regrowth, the induced spores and spores developed in fruiting bodies were shaken with 0.1% Triton X-100, washed and plated on 1/5 ^th^ SM plates (14 cm Ø) with *K. aerogenes* at 500 cells/plate and emerging plaques were counted after 4 days at 21°C. As the recovery of growing amoebas from induced spores was poor, they were plated at 2,000 cells/plate in subsequent experiments.

### Data analysis

For phylogenetic inference, protein sequences were aligned with Clustal Omega ( Sievers & Higgins, 2014) (RRID:SCR_001591) and phylogenetic trees were inferred with MrBayes 3.2 ( Ronquist & Huelsenbeck, 2003) (RRID:SCR_012067) using a mixed amino acid model. Trees were annotated with protein functional domain architectures as analysed with SMART ( Schultz *et al.*, 1998) (RRID:SCR_005026).

Experimental data were compiled in Microsoft Excel version 2108 (RRID:SCR_016137) for basic calculations and descriptive statistics (Means and SD). SigmaPlot v14.5 (RRID:SCR_003210; Systat Software, Inc.) was used to assess significant differences between measured parameters. For comparison between two datasets a t-test was used when the data were normally distributed and a rank sum test when they were not. For comparisons between three or more datasets one-way analysis of variance (ANOVA) or ANOVA on ranks were used.

## Results

### Identification, expression and disruption of *P. pallidum* autophagy genes

**Figure 2.**
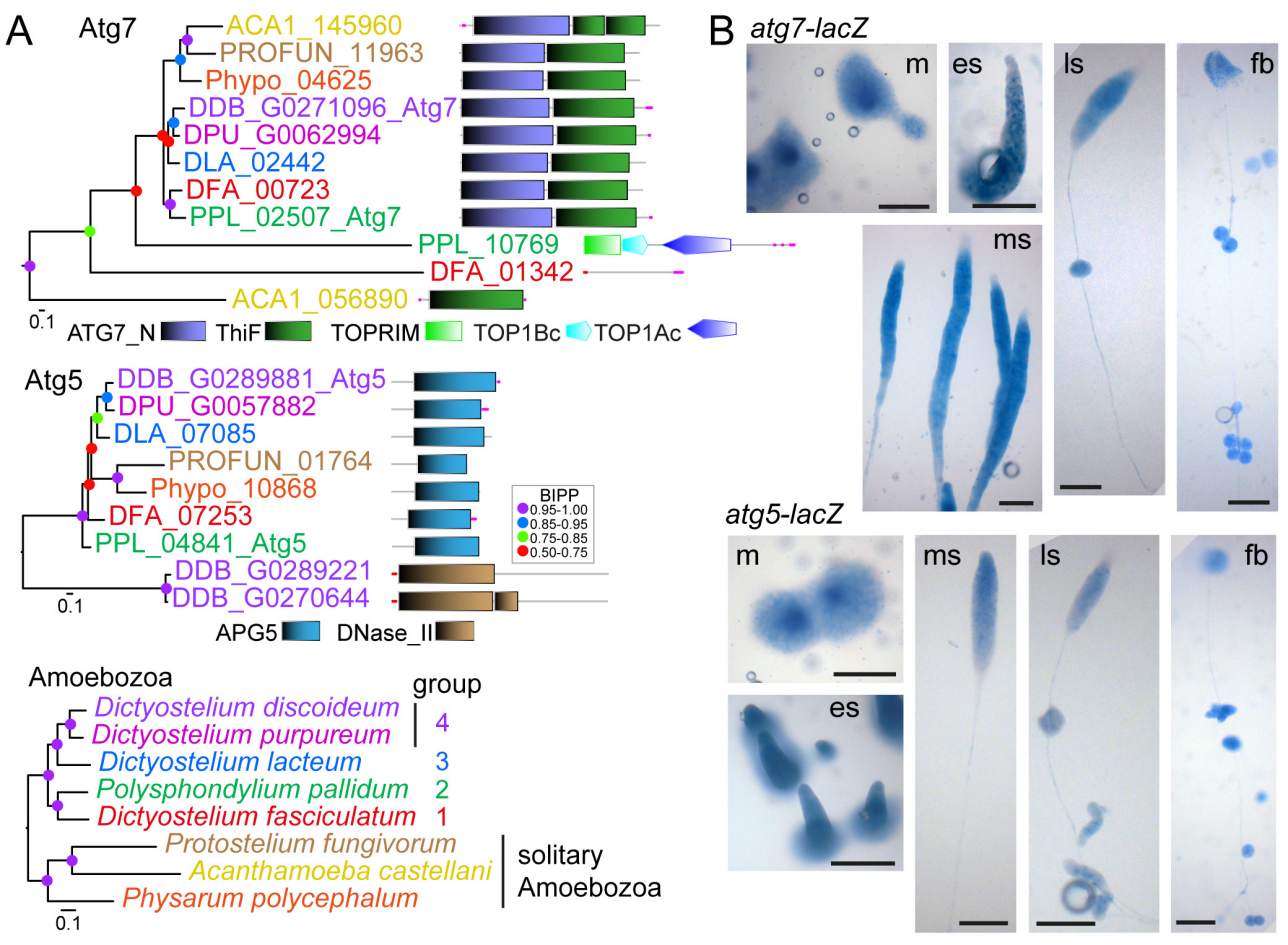
Identification and expression patterns of *P. pallidum* strain PN500_J *(Ppal) atg7* and *atg5.* ***A***. *Identification.* The previously identified *Dictyostelium discoideum* ( *Ddis*) *atg7* and *atg5* genes ( Otto *et al.*, 2003) were used as bait to identify homologs in well-annotated dictyostelid and solitary amoebozoan genomes. Protein sequences of all hits were aligned with the *Ddis* sequences and the alignment was used to infer phylogenies using MrBayes 3.2 ( Ronquist & Huelsenbeck, 2003). Posterior probabilities of the nodes (BIPP) are indicated by coloured dots. Trees were annotated with the functional domain architecture of the proteins as analysed with SMART ( Schultz *et al.*, 1998). A multigene amoebozoan phylogeny ( Schilde *et al.*, 2019) is shown as reference. ***B***. *Expression. Ppal* cells, transformed with gene fusions of the *Ppal atg7* or *atg5* promoters and *Escherichia coli lacZ,* were plated on nitrocellulose filters supported by non-nutrient (NN) agar. Developing structures were fixed in glutaraldehyde and stained with X-gal ( Dingermann *et al.*, 1989). m: mound; es, ms and ls: early, mid- and late sorogens; fb: fruiting body. Bar: 100 µm.

To investigate evolutionary conservation of the role of autophagy in dictyostelid sporulation and possible involvement in encystation, we searched for orthologs of the essential autophagy genes a *tg7* and *atg5* in in taxon group representative dictyostelid genomes and three genomes of solitary Amoebozoa. Sequence alignments were used to infer phylogenetic trees, which were annotated with the functional domain architecture of the proteins ( Figure 2A) ( Schaap, 2022). Most of the Atg7 and Atg5 homologs combined into a single clade each and contained an ATG7_N or an APG5 domain, respectively. Some more distantly related homologs did not contain these domains. The analysis identified PPL_02507 and PPL_04841 as *Ppal* Atg7 and Atg5, respectively, and highlighted that *atg7* and *atg5* are conserved as single copy genes throughout Amoebozoa. To investigate the expression pattern of either gene, we transformed *Ppal* with a fusion construct of their promoter and the *LacZ* reporter and stained developing structures with *Xga*l. Both *atg7* and *atg5* were expressed throughout multicellular development, with reduced expression of *atg7* in the tips of emerging sorogens ( Figure 2B).

**Figure 3.**
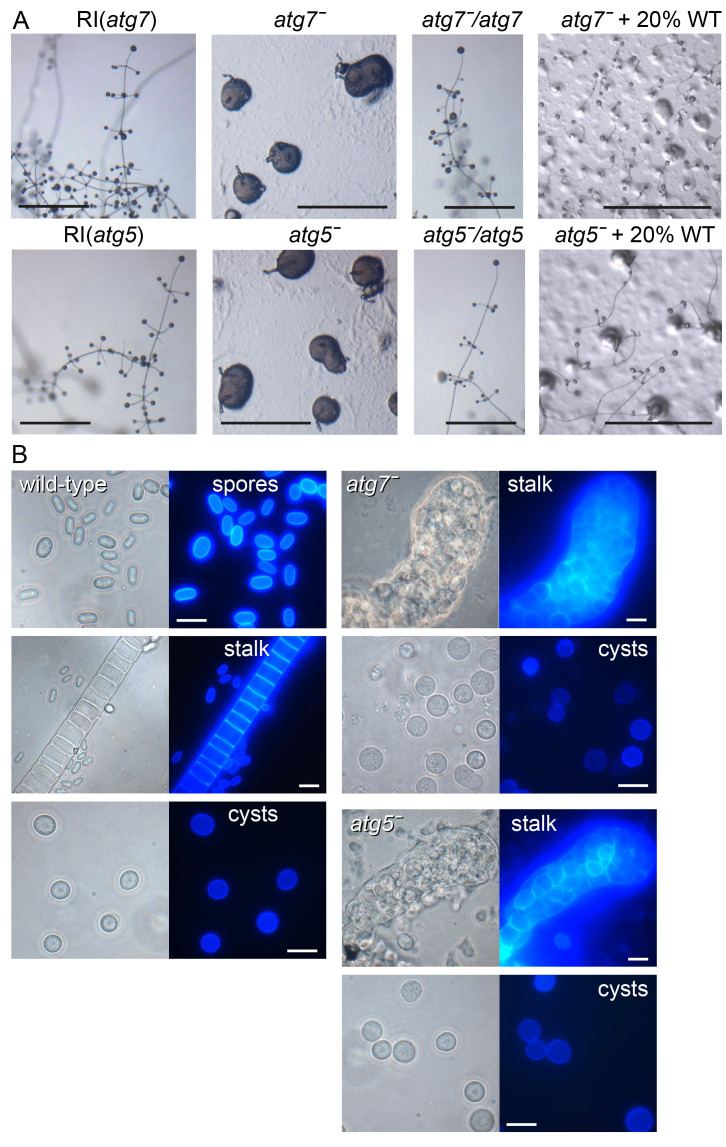
Phenotypes of *P. pallidum* strain PN500_J ( *Ppal*) *atg7* and *atg5* mutants. ***A***. *Ppal atg7ˉ* and *atg5ˉ* knock-outs, random integrant (RI) and *atg7ˉ* and *atg5ˉ* cells complemented with *atg7* or *atg5*, respectively, or mixed with 20% wild-type (WT) were plated on non-nutrient (NN) agar and incubated until mature terminal structures had formed. Bar: 100 µm. ***B***. Terminal structures of WT, *atg7ˉ* and *atg5ˉ* were transferred to 0.001% Calcofluor on a slide glass. Stalks, spores and cysts were photographed under phase contrast (left panels) and UV (right panels). Bar: 10 µm.

We next generated lesions in the *Ppal atg7* and *atg5* genes by homologous recombination. The KO constructs contained the loxP-neo selection cassette flanked by two ~1kb fragments of the *atg7* or *atg5* genes and transformation into *Ppal* yielded several KO and random integrant (RI) clones for each construct ( Figure 1). KO, RI and WT *Ppal* cells were developed into multicellular fruiting bodies on agar plates and into unicellular cysts in suspension. Mature cells and structures were stained with Calcofluor to visualize cellulose cell walls ( Figure 3).

The RI clones showed the same fruiting bodies as WT *Ppal*, but both the *atg7ˉ* and *atg5ˉ* KO clones showed severe defects. On agar, amoebas aggregated normally, and tips appeared on the aggregates ( Figure 3A). However, most of the cell mass was never lifted off the substratum. Instead, small finger-like projections were formed that consisted almost entirely of vacuolated stalk-like cells, with cells in the basal mass remaining amoeboid. No spores differentiated at all, and the stalk-like structures were thicker and more irregular than WT stalks ( Figure 3B). To confirm that the phenotypic abnormalities were due to loss of the *atg7* and *atg5* genes, we removed the loxPneo cassette from the *atg7ˉ* and *atg5ˉ* mutants by transformation with cre-recombinase and transformed each mutant with its missing gene expressed from its own promoter. Both the *atg7ˉ/atg7* and the *atg5ˉ/atg5* mutants reverted to the normal WT phenotype ( Figure 3A). To investigate whether the *atg7ˉ* and *atg5ˉ* defects were cell-autonomous, we mixed either mutant with 5% or 20% WT cells. Only in the mixture with 20% WT were some small fruiting bodies detected. However, none of their spores were G418 resistant, indicating that neither *atg7ˉ* nor *atg5ˉ* had formed spores in the chimeras. Their sporulation defect is therefore cell-autonomous.

**Figure 4.**
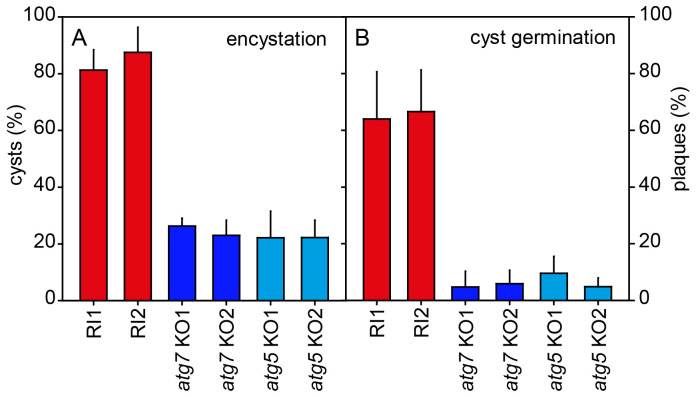
Encystation and cyst viability. **A**. *Encystation.* Random integrant (RI) and two knock-out (KO) clones each of *atg7ˉ* and *atg5ˉ* were harvested from growth plates and incubated in 400 mM sorbitol in KK2 for three days. After addition of Calcofluor to 0.001%, total cells and fluorescent cysts were counted and the percentage of encysted cells was calculated. Means and SD of three experiments. **B**. *Cyst viability*. Cysts induced for four days, as described above, were treated with 0.1% Triton X-100 to lyse unencysted amoebas and then distributed on growth plates together with *K. aerogenes* at 500 cells/plate (Ø 14 cm). After four days of growth at 22°C, the emerging plaques were counted and calculated as percentage of the plated cells. Means and SD of two experiments with duplicate plates for each cell line. For both experiments the individual RI values were significantly different from the individual KO values at P<0.001 as tested by one-way ANOVA.

When starved in suspension at high osmolarity, all WT and *atg7ˉ* and *atg5ˉ* amoebas assumed the rounded cyst morphology ( Figure 3B). However, compared to WT cells, fewer of the rounded *atg7ˉ* and *atg5ˉ* cells developed Calcofluor positive cell walls, suggesting that the encystation process was incomplete. Quantitation of the number of Calcofluor positive cysts formed by different KO and RI clones showed that after three days in encystation medium, over 81–87% of RI cells had formed Calcofluor positive cysts, while this was only the case for 22–26% of *atg7ˉ* or *atg5ˉ* KO cells ( Figure 4A).

To test whether loss of *atg7* or *atg5* also affected cyst viability. RI and *atg7ˉ* and *atg5ˉ* cells, incubated for four days in encystation medium, were treated with 0.1% Triton-X100 to lyse unencysted amoebas and then counted and plated together with *Klebsiella aerogenes* on growth plates. Of the plated RI cysts 64–66% formed plaques of feeding amoebas, but this was only the case for 3–10% of *atg7ˉ* or *atg5ˉ* cysts ( Figure 4B). Apparently, loss of *atg7* or *atg5* reduced the ability of amoebas to encyst and the cysts that were formed were less viable.

### *Ppal atg7 ^-^* and *atg5 ^-^* mutants show defective prespore gene expression

To gain insight into the cell differentiation anomalies of the *atg7ˉ* and *atg5ˉ* mutants, we measured expression of the *Ppal* prespore gene *sp45* (PPL_06034) and the prestalk gene PPL_04427 by RT-qPCR. Sp45 is a member of the spore coat (Cot) family of proteins ( Fosnaugh *et al.*, 1994) that typically harbour a signal peptide and Follistatin-N-terminal (FOLN) repeats. Its prespore-specificity was demonstrated by *in situ* hybridization and expression of GFP from the *sp45* promoter ( Gregg & Cox, 2000). PPL_04427 is a close relative of the *Ddis* prestalk/stalk markers *ecmA* and *ecmB* and its specificity for *Ppal* prestalk and stalk cells was shown by expression of *LacZ* from the PPL_04427 promoter ( Schilde *et al.*, 2014). To use as controls for standardization in qPCR we sought out well-expressed genes that were constitutively expressed in *Ppal* development in three RNAseq experiments. Because mature cell types in *Ppal* are either dormant or dead, there are only few candidates (SupdataRNAseq.xlsx, available as *Underlying data* ( Schaap, 2022)). We selected PPL_07209, which also showed a reasonable level of read counts.

**Figure 5.**
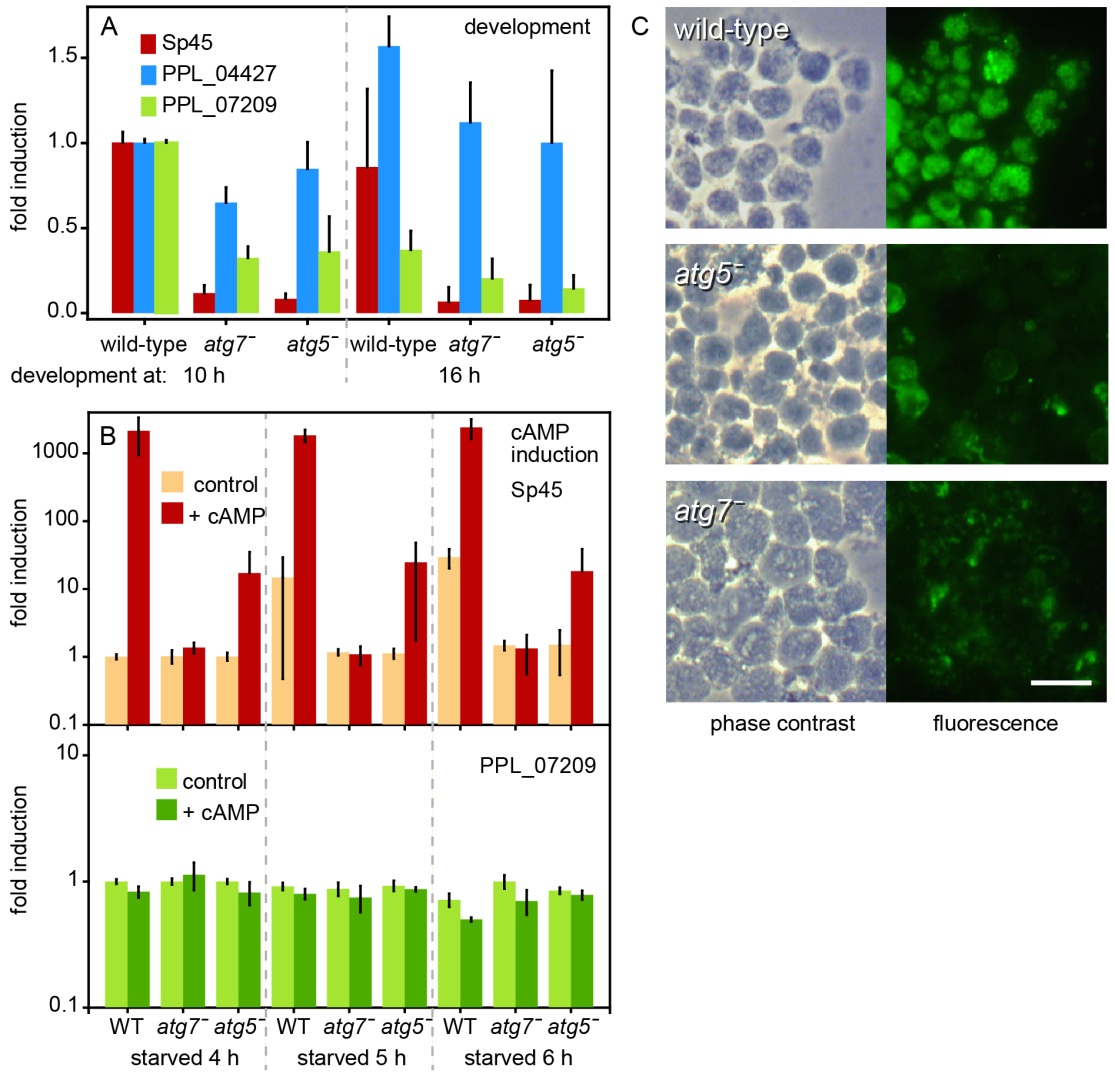
Developmental and induced gene expression. ***A***. *Developmental expression. P. pallidum* strain PN500_J *(Ppal)* wild-type (WT) cells and *atg7ˉ* and *atg5ˉ* mutants were developed on non-nutrient (NN) agar for 10 h and 16 h, when mid and late sorogens had formed. RNA was isolated and the expression of the prespore gene *sp45*, the prestalk gene *PPL_04427* and the constitutive gene *PPL_07209* were probed by reverse transcription-quantitative (RT-q)PCR. Data for each gene were normalized to expression in WT structures at 10 h. Means and SD of three experiments assayed with technical duplicates. ***B***. *cAMP induction of sp45.* WT cells and *atg7ˉ* and *atg5ˉ* mutants were starved on agar for 4, 5 and 6 h until loose to tight aggregates had formed. Aggregates were dissociated and cells were incubated in suspension with and without 1 mM cAMP for 4 h. RNA was isolated and expression of *sp45* and *PPL_07209* was probed by RT-qPCR. Data were normalized to expression in 4 h starved WT cells, incubated for 4 h without cAMP and Sp45 data were standardized on expression of PPL_07209 in the same sample. Means and SD of three experiments assayed with technical duplicates. ***C***. *Spore antigens.* WT and *atg5ˉ* and *atg7ˉ* mutants were developed for 15 h into sorogens, which were dissociated and stained with anti-spore antibodies and Alexa fluor 488 goat-anti-rabbit-IgG. Bar: 10 μm.

To compare prestalk and prespore gene expression in stages where WT *Ppal* forms mid- and late sorogens, we developed WT, *atg7ˉ* and *atg5ˉ* cells for 10 and 16 h on NN agar. RNA was isolated and reverse-transcribed and qPCR was performed on 60 ng cDNA with the primers listed in Table 1. Data for individual genes were normalized to Cq values obtained from WT samples at 10 h of development. Expression of the *sp45* prespore gene was about 10-fold lower in *atg7ˉ* and *atg5ˉ* structures than in WT sorogens and showed no significant increase between 10 and 16 h ( Figure 5A). The prestalk gene *PPL_04427* increased at 16 h to 1.5x the level at 10 h and was at either time point about 20–30% lower in *atg7ˉ* and *atg5ˉ* than in WT. The "constitutively" expressed gene *PPL_07209* decreased both between 10 h and 16 h in WT and in *atg7ˉ* and *atg5ˉ,* compared to WT. Standardization of the *sp45* and *PPL_04427* data on *PPL_07209* massively inflated *PPL_04427* expression. Since the RT-qPCR reactions are in effect already standardized by using the same amount of template as input and showed little variation between three individual experiments and between *atg7ˉ* and *atg5ˉ*, we consider the uncorrected data in Figure 5A to reflect the relative expression levels of *sp45* and *PPL_04427* more accurately.

Prespore differentiation in both *Ddis* and *Ppal* requires stimulation of cell surface cAMP receptors (cARs) by secreted cAMP ( Kawabe *et al.*, 2009; Schaap & Van Driel, 1985; Wang *et al.*, 1988). The diminished *Sp45* expression and lack of spore differentiation in *atg7ˉ* and *atg5ˉ* mutants, prompted us to examine whether prespore gene induction by cAMP was impaired. *Ppal* cells were starved on NN agar for 4, 5 and 6 h, when loose to tight aggregates have formed, to reach a stage where cells are competent for prespore gene induction but have not started to express prespore genes. The aggregates were dissociated and cells were incubated for 4 h in suspension with 1 mM cAMP. RNA was isolated and expression of *sp45* and *PPL_07209* was determined by RT-qPCR. Data were normalized to *sp45* or *PPL_07209* Cq values in 4 h starved WT cells that were incubated without cAMP. In this experiment the PPL_07209 Cq values were almost unchanged between WT and mutant cells and between treatments ( Figure 5B) and were therefore used to standardize the *sp45* fold-change induction. In WT, cAMP increased *sp45* expression over 2000-fold in 4 h starved cells. In 5 h and 6 h starved cells unstimulated *sp45* expression was 15–30-fold higher than in 4 h starved cells, while cAMP-induced levels were the same as in 4 h starved cells. cAMP induction of *sp45* was absent in *atg7ˉ* and reduced from 2,000 to 20-fold in *atg5ˉ.* Evidently, as is the case in *Ddis* ( Yamada & Schaap, 2019), cAMP induction of prespore gene expression in *Ppal* requires autophagy genes.

Spore coat proteins such as Sp45 are in Dictyostelia synthesized at the inner membrane of Golgi-derived prespore vesicles. These vesicles are exocytosed during spore maturation bringing the first layer of the spore coat to the surface of the spore ( West, 2003). Antibodies generated against intact spores have been widely used to visualize the prespore vesicles inside prespore cells ( Schilde *et al.*, 2014; Takeuchi, 1963). To appreciate the extent of the sporulation defect, we stained dissociated maturing sorogens of WT, *atg7ˉ* and *atg5ˉ* mutants with antibodies against a mixture of *Ddis* and *Ppal* spores ( Schilde *et al.*, 2014) to evaluate the presence of cells with prespore vesicles. Figure 5C shows that WT *Ppal* cells contained many vesicles lined with spore antigens. While *atg5ˉ* and *atg7ˉ* cells showed some reactivity to spore antibodies, this was mostly localized between cells or on the cell surface and is likely non-specific.

Together with the lack of prespore gene expression and induction in *Ppal atg5ˉ* and *atg7ˉ*, these data show that autophagy is required in *Ppal* for prespore gene induction by cAMP and spore differentiation.

### Size and viability of spores formed in- and outside multicellular structures

As argued above, loss of autophagy is likely to affect spore viability. Without it, the starving cells would not be able to generate the compounds required for spore wall synthesis and energy storage. It is however unclear why loss of autophagy should specifically act at initial induction of prespore gene expression by cAMP. Autophagy, as measured by RFP-GFP-Atg8 containing vesicles ( Yamada & Schaap, 2021) or the percentage of cytosol occupied by autophagosomes ( Schaap, 1983), is much higher in prestalk than in prespore cells. Our present observation that encystation is less affected than sporulation by loss of autophagy, combined with low autophagy in prespore cells suggests that in multicellular development the spores depend on metabolites produced by autophagy in the prestalk/stalk population.

**Figure 6.**
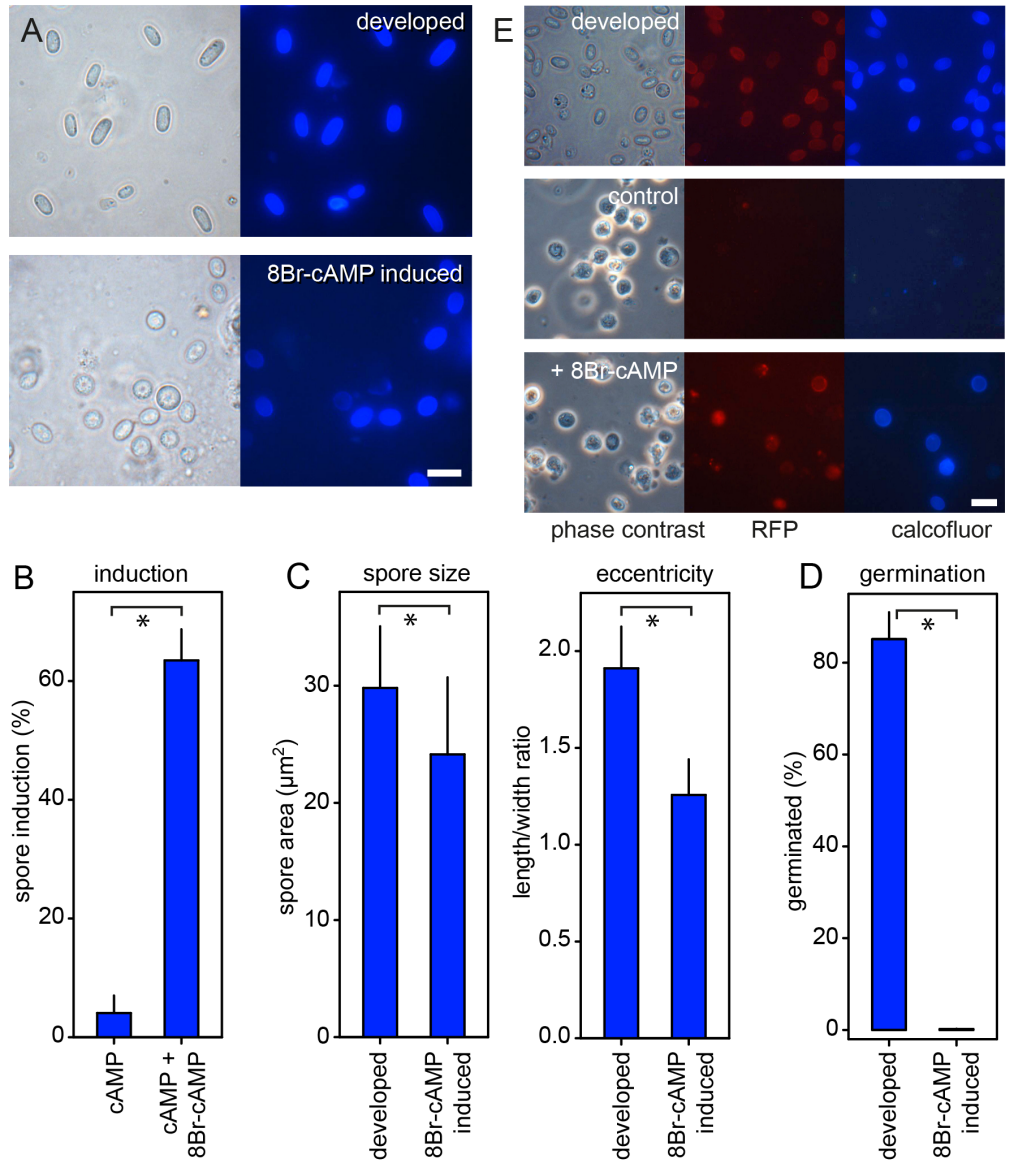
Features of naturally developed and 8Br-cAMP-induced *Dictyostelium discoideum* ( *Ddis*) spores. ****A***. Microscopy*. Wild-type (WT) *Ddis* Ax2 spores were isolated from mature fruiting bodies (developed) and harvested after 30 h of incubation with 5 mM cAMP and 15 mM 8Br-cAMP. The spores were stained with 0.001% Calcofluor and imaged under phase contrast and epifluorescence. Bar: 10 μm. ***B***. *Quantitation. Ddis* cells were incubated for 30 h with 5 mM cAMP with and without 15 mM 8Br-cAMP, stained with Calcofluor. The numbers of total and Calcofluor stained cells were determined from phase contrast and fluorescence images, and the percentage of Calcofluor positive cells (spores) was calculated. Means and SD of three experiments. ***C***. *Size and eccentricity.* The displayed area and the spore length and width of Calcofluor positive spores were measured using ImageJ ( Collins, 2007) and spore eccentricity (length/width) was calculated. Means and SD of 100 spores for each condition are shown. ***D***. *Germination.* Developed and induced spores were treated with 0.1% Triton to lyse unencapsulated amoebas, counted and plated with *K. aerogenes* at 500 cells/plate for developed and 2,000 cells/plates for induced spores. After three days the emerged plaques were counted, and the percentage of germinated spores was calculated. Means and SD of three independent experiments. *Significant differences (P<0.001) between the two datasets presented in each graph were determined by a rank sum test. ***E***. *Cotc-mRFP expression. Ddis* Ax2 cells, transformed with a gene fusion of the mRFP gene and *Ddis* spore coat gene *cotC* inclusive of its promoter were developed into spores in fruiting bodies (top row) or incubated for 30 h with 5 mM cAMP (control) or 5 mM cAMP and 15 mM 8Br-cAMP. The cells were stained with Calcofluor and imaged in phase contrast and by epifluorescence. Bar: 10 μm.

In *Ddis,* spores can be induced to differentiate from single amoebas in suspension by treatment with ≥10 mM 8Br-cAMP, a membrane-permeant PKA agonist, although the efficiency of induction varies between different strains ( Kay, 1989; Richardson *et al.*, 1991). In *Ppal,* 8Br-cAMP effectively and invariably induces encystation ( Ritchie *et al.*, 2008). Because group four species like *Ddis* have lost encystation ( Romeralo *et al.*, 2013), we used *Ddis* to compare the size and viability of spores developed inside the fruiting body with those of spores induced in cell suspension by 8Br-cAMP. To increase the efficiency of sporulation, we plated cells at a density of 10 ^5^ cells/cm ^2^, which allows some cell clumping and interaction of the tgrB1/tgrC1(lagC) adhesion proteins that induce competence for post-aggregative gene induction ( Dynes *et al.*, 1994; Hirose *et al.*, 2011). Pilot experiments showed that spore induction was also improved when 5 mM cAMP was included with 15 mM 8Br-cAMP, but cAMP induced little spore encapsulation by itself ( Figure 6B).

About 64% of *Ddis* Ax2 amoebas incubated with 5 mM cAMP and 15 mM 8Br-cAMP differentiated into ellipsoid spores with cellulose-rich walls, as evaluated by staining with Calcofluor ( Figure 6A and B). The eccentricity (length to width ratio) of the induced spores was 34% lower than that of spores developed in fruiting bodies, and their cross-section area was reduced by 20% ( Figure 6C). The viability of 8Br-cAMP induced spores is usually assessed by their resistance to lysis by detergents ( Kay, 1989). While the induced spores in our experiments were not lysed by detergent treatment, only 0.2% of the detergent-treated spores germinated when plated with bacteria on agar. For developed spores 85% germinated after detergent treatment ( Figure 6D).

To confirm that the encapsulated cells were spores, we repeated the induction by 8Br-cAMP using *Ddis* expressing the spore coat gene *cotC* fused to red fluorescent protein (mRFP) ( Yamada *et al.*, 2018). While this strain showed less efficient induction of Calcofluor positive cells (17%) than its parent Ax2, most Calcofluor positive cells also showed mRFP expression indicating that they had entered the sporulation pathway ( Figure 6E). In conclusion, compared to spores developed in the presence of prestalk and stalk cells, spores induced by 8Br-cAMP in isolation were rounder, smaller and non-viable after detergent treatment.

## Discussion

### Multicellular sporulation depends more on autophagy than unicellular encystation

Autophagy is very prominent in the prestalk and stalk population of *Ddis* and was initially considered to be required for stalk cell differentiation ( Giusti *et al.*, 2009; Schaap, 1983). However, a screen for sporulation-deficient mutants identified lesions in several autophagy genes as the cause of the sporulation defects. Such mutants had relatively normal stalks and overproduced the stalk-like basal disc cells but were specifically defective in cAMP-induced prespore gene expression ( Yamada & Schaap, 2019; Yamada & Schaap, 2021).

To investigate whether this specific effect of autophagy is conserved in Dictyostelia and whether autophagy is also required for encystation, the ancestral survival strategy of solitary amoebas, we deleted two genes, *atg7* and *atg5* that are each essential for early autophagosome formation in *Ppal*, a distant relative of *Ddis* that can form both spores and cysts. The *Ppal atg7ˉ* and *atg5ˉ* mutants showed similar defects in multicellular development as their *Ddis* counterparts. They normally aggregated into mounds, but never formed normal sorogens (or slugs in *Ddis*). Instead, small finger-like structures projected from the mounds that consisted mostly of stalk cells, while the remaining cells stayed amoeboid ( Figure 3).

No spores were formed at all and the amoeboid cells did not contain the characteristic prespore vesicles with spore antigens. Expression of the prespore gene *sp45* was much reduced in the *atg7ˉ* and *atg5ˉ* multicellular structures. Prespore gene induction by cAMP was absent in *atg7ˉ* and 100-fold reduced in *atg5ˉ* cells. When starved as single cells in suspension *Ppal atg7ˉ* and *atg5ˉ* amoebas did form cysts, but the percentage of properly walled cysts was 3-fold lower than for WT and only 5–10% of the mutant cysts germinated into viable amoebas. Both encystation and sporulation are a response to starvation stress and it takes about 20-24 h for mature cysts or spores to form. Both require cell wall synthesis and likely deposition of energy stores for the emerging amoebas. It therefore stands to reason that the differentiation of viable spores or cysts requires turnover of existing macromolecules and organelles by autophagy. It is however unclear why this requirement is much more stringent for sporulation and why loss of autophagy should so specifically act on cAMP induction of prespore gene expression. Additionally, it is unclear why stalk cell differentiation, which involves fusion of acidic vacuoles into a large central vacuole and cell wall synthesis, does not require autophagy. In *Ddis atg7ˉ* and *atg5ˉ* mutants, the prespore population transdifferentiates into the stalk-like basal disc cells. This does not occur in *Ppal,* presumably because basal disc cells are a group 4 specific innovation ( Romeralo *et al.*, 2013). Recently, *Ddis* mutants lacking PIKfyve, a 1-phosphatidylinositol-3-phosphate 5-kinase, which is involved in fragmentation of late (auto)-lysosomes during normal endosome processing, were also shown to transdifferentiate prespore cells into basal disc cells ( Yamada *et al.*, 2021). Together with the phenotypes of the *atg7ˉ* and *atg5ˉ* mutants, this suggest that the extreme vacuolation and lysis of cell content that accompanies basal disc differentiation represents a dysregulation of lysosomal function rather than a dependence on autophagy.

### cAMP as a signal for the aggregated state

In both *Ddis* and *Ppal*, prespore differentiation is induced by micromolar cAMP acting on cAMP receptors ( Kawabe *et al.*, 2009; Schaap & Van Driel, 1985) and also requires activation of PKA by increased intracellular cAMP ( Hopper *et al.*, 1993; Mann *et al.*, 1994). The effect of PKA is however not specific for spore differentiation, since stalk cell differentiation also requires PKA ( Harwood *et al.*, 1992) and PKA activation is the only signal required for encystation ( Kawabe *et al.*, 2015). In Dictyostelids and solitary Amoebozoa stressors like starvation and high osmolarity increase intracellular cAMP, which by acting on PKA then induces encystation. This indicated that the role of PKA in spore and stalk cell encapsulation is evolutionary derived from its role in encystation ( Du *et al.,* 2014; Ritchie *et al.*, 2008).

This notion was supported by the finding that *Ppal* cAR KOs formed cysts in their fruiting bodies instead of spores. The cAR KOs had lost cAMP induction of prespore gene expression, but since cAMP levels and therefore PKA activity were still elevated in the starving cells, encystation was their remaining option ( Kawabe *et al.*, 2009). Because Dictyostelids secrete most of the cAMP that they synthesize, it was hypothesised that accumulation of micromolar extracellular cAMP within aggregates acts as a signal for the aggregated state, inducing *Ppal* cells to form spores and not cysts when in aggregates.

This raised a further question into the fitness advantage of spores over cysts. Experiments measuring ultrastructural features and testing long term survival of spores over cysts showed that while spores and cysts of the same species showed similar long-time survival at 22°C, spores survived frost much better, which was correlated with spores having a thicker more structured cell wall and higher state of dehydration than cysts ( Lawal *et al.*, 2020). Compared to spores from groups one, two and three, group four spores combined the thickest cell walls with large spore size and high dehydration and showed the highest frost resistance. In contrast to species from groups one to three, which were mainly isolated from tropic to temperate zones, group four contains many species that were isolated from arctic and alpine regions, suggesting that the improved frost survival of their spores allowed group four species to colonize colder habitats ( Lawal *et al.*, 2020). How is this related to autophagy?

### Are prestalk and stalk cells nursing the spores?

*Ddis* prestalk cells typically contain more neutral red stained acidic vesicles than prespore cells ( Devine & Loomis, 1985) as well as vesicles with autophagy proteins like Atg8, KnkA and Bcas3 ( Yamada & Schaap, 2021), indicating that autophagy is normally more active in prestalk than in prespore cells. To test a hypothesis that sporulation directly benefits from prestalk cell autophagy, we compared the overall size and fitness of *Ddis* spores formed in fruiting bodies with those induced to sporulate as solitary cells with 15 mM 8Br-cAMP and 5 mM cAMP. The induced spores were smaller and rounder than the spores from fruiting bodies and while they incorporated both spore coat proteins and cellulose in their cell walls, they lost the ability to germinate and resume feeding after detergent treatment, which is not the case for WT spores ( Figure 6).

While other explanations like missing signals for full spore maturation remain possible, it is most likely that full spore maturation requires flow of metabolites from the prestalk/stalk to the prespore population. In such a scenario the dependence of prespore gene expression on micromolar extracellular cAMP that can only occur when cells are close together in aggregates, combined with the dependence of cAMP signal transduction on autophagy signifies that the starving amoebas use cAMP to test whether they are in a state (the aggregate) where they can benefit from the autophagy of others. If, as is the case in *Ppal* cAR KOs, the cAMP increase cannot be sensed, the cells opt to differentiate into the less resilient cysts ( Kawabe *et al.*, 2009).

### Exploitation of the weak analogies between spores and sexual macrocysts

Sexual macrocysts are another heavily walled survival structure of Dictyostelia, which typically maintain dormancy for very long periods. Here, two starving cells of opposite mating type fuse and the zygote then secretes chemoattractant to lure other amoebas into an aggregate. The zygote then cannibalizes these amoebas and uses their metabolites to build its heavy wall ( O'Day & Keszei, 2012).

The reliance of spores on nutrients from prestalk/stalk cells is analogous to the reliance of zygotes on co-aggregated haploid cells and provides insight into the early evolution of somatic cells. While starving proto-dictyostelia may originally have aggregated to protect their dormant cells from predation by larger starving protists, as was demonstrated for the Volvocales ( Herron *et al.*, 2019), the aggregated cells then exploited each other to improve their long-term survival. For the zygote, feeding on other amoebas is facilitated by it being twice as large. For asexual aggregates it is well-documented that cells that enter starvation while late in the cell cycle (and are thus relatively large) or cells fed in glucose-rich *versus* glucose-poor media preferentially differentiate into spores compared to cells that have just divided ( Gomer & Firtel, 1987; Leach *et al.*, 1973; Ohmori & Maeda, 1987; Weijer *et al.*, 1984). The differentiating prespore cells then secrete compounds, such as DIF-1, that prevent other cells from differentiating as prespore cells, but to which they are less responsive ( Kay & Thompson, 2001; Thompson & Kay, 2000). In short, the larger well-fed cells are predisposed to propagate the organism and then coerce the leaner cells to give up their resources through autophagy.

### Size and complexity of soma correlates with improved hibernation

In *Ddis* and other group four species, prestalk cells occupy the anterior 20–30% of the sorogen, while the posterior prespore cells maintain a proportion of anterior-like cells by secretion of DIF-1 and other factors ( Kay *et al.*, 1999). Some anterior-like cells replenish the prestalk cells during formation of the stalk, which is several cells thick, while others either differentiate into basal disc or cup cells that respectively support the stalk and spore mass ( Sternfeld, 1998; Sternfeld & David, 1982).

In groups one to three, stalks are one cell thick and the prestalk region only makes up the anterior 5–10% of the sorogen ( Gregg & Cox, 2000). Prespore cells transdifferentiate into prestalk cells at this region, but scattered expression of (pre)stalk markers throughout *Ppal* sorogens suggests that group one to three species may have anterior-like cells ( Schilde *et al.*, 2014). Even if so, the ratio of somatic over spore cells is much lower in groups one to three than in group four and there is only one somatic cell type, the stalk cell.

As mentioned above, compared to group one to three spores, group four spores are more dehydrated and have thicker cell walls, factors that likely assist their surviving longer under frosty conditions than group one to three spores, and to group four species being common to arctic and alpine regions, where group one to three species are rarely found ( Lawal *et al.*, 2020). Fossil calibrated phylogenies date the split between the two major branches of Dictyostelia at 0.52 bya, just following the global Neoproterozoic glaciations. Because cysts combine good long-time survival above 20°C with poor frost survival, it was surmised that sporulation in multicellular fruiting bodies evolved in response to global cooling ( Lawal *et al.*, 2020). Partitioning ever larger numbers of cells to somatic fate, allowing increased nutrient flux to spores by autophagy, may have allowed group four to further increase the cold resistance of its spores and to inhabit the coldest regions of the planet. The increased size of the somatic cell pool also allowed the somatic cells to assume novel roles as basal disc and cup cells.

Additional work is needed to support this narrative. Spore wall thickness and spore compaction were relatively easy to determine for many species by electron microscopy ( Lawal *et al.*, 2020). However, spore fitness likely involves other factors that require more in-depth experimentation, such as spore wall composition and architecture as well as the size of their trehalose and lipid stores. Particularly trehalose, which accumulates in maturing spores ( Rutherford & Jefferson, 1976) and acts both as an energy store utilized during spore germination ( Jackson *et al.*, 1982) and as a cryo- and desiccation protectant of proteins and membranes ( Elbein *et al.*, 2003) may be a major determinant for long term spore survival.

The role of sexual macrocysts in dictyostelid survival in different ecological niches is unknown. They are formed under dark and submerged conditions and induced by ethylene ( Amagai *et al.*, 2007). Unlike spores and cysts, macrocysts require long periods and as yet unknown stimuli to germinate. The latter property prohibited experimental studies, although progress was made in identification of genes required for macrocyst formation and genes that define the three mating types of *Ddis* ( Bloomfield, 2019; Urushihara & Muramoto, 2006). Macrocysts are common throughout the dictyostelid phylogeny ( Schaap *et al.*, 2006) and their prolonged dormancy suggests a major role in long-term stress survival.

However, while the cannibalism that feeds the macrocyst seems an evolutionary dead-end towards developing multicellular complexity, autophagy as occurs in fruiting bodies does not exclude additional functionality of the soma. Autophagy is an ancient and well-conserved process across all eukaryote divisions ( Zhang *et al.*, 2021), with most divisions also giving rise to multicellular forms ( Brown *et al.*, 2012). The current work indicates that autophagy may have played a major role in the initial evolution and diversification of somatic cells.

## Data availability

### Underlying data

NCBI Gene: Heterostelium album strain PN500_J autophagy protein 7 (atg7) gene, complete cds. Accession number ON758339, https://www.ncbi.nlm.nih.gov/nuccore/ON758339

NCBI Gene: Cloning vector EXP5(+), complete sequence. Accession number EF028664.1, https://www.ncbi.nlm.nih.gov/nuccore/EF028664.1/

Open Science Framework: ExtendedData_Du_MS. https://doi.org/10.17605/OSF.IO/XCAZG ( Schaap, 2022).

This project contains the following underlying data:

- SupdataRnaSeq.xlsx
- DNAconstructMaps_Sequences.zip (DNA constructs, gene sequences and plasmid maps)
- Fig1_uncropped_gel_images.zip (Uncropped gel images for Figure 1)
- atg5_atg7-lacZstaining_originals.zip (atg5-atg7 promoter_LacZ staining original images)
- atg5_7KOphenotype_originalimages.zip (atg5-atg7 KO phenotype original images)
- statistics.zip (Raw spreadsheet data of statistical analyses performed)
- RTqPCRexpCqvalues+calculation.zip (Standard curves and raw Cq values for all samples and replicates (qPCR))
- Spore-antibodyStaining_Originalimages.zip (Spore antibody staining original images)
- 8BrcAMPspore-Induction_original_images.zip (8Br-cAMP induced spores original images)

Data are available under the terms of the Creative Commons Zero "No rights reserved" data waiver (CC0 1.0 Public domain dedication).

## Ethics and consent

Ethical approval and consent were not required.
