# Supplementary material for: Autophagy of the somatic stalk cells likely nurses the propagating spores of Dictyostelid social amoebas
Source: Open Res Eur. 2022 Nov 23;2:104. Originally published 2022 Sep 1. [Version 2] doi: 10.12688/openreseurope.14947.2 (PMC7614253; doi:10.12688/openreseurope.14947.2)
Supplement: Peer review history

# Peer review: Reviewer response for version 2

**DOI:** 10.21956/openreseurope.16588.r30414

**Published:** 2022-11-29

**Authors:** Ricardo Escalante

**Affiliations:** 1 C.S.I.C./U.A.M, Instituto De Investigaciones Biomédicas Alberto Sols, Madrid, Spain

The authors responded satisfactorily to all my concerns. I have no further questions. This manuscript is an important contribution to the field.

Is the study design appropriate and does the work have academic merit?

Yes

Is the work clearly and accurately presented and does it cite the current literature?

Yes

If applicable, is the statistical analysis and its interpretation appropriate?

Partly

Are all the source data underlying the results available to ensure full reproducibility?

Yes

Are the conclusions drawn adequately supported by the results?

Partly

Are sufficient details of methods and analysis provided to allow replication by others?

Yes

Reviewer Expertise:

Autophagy; Dictyostelium

I confirm that I have read this submission and believe that I have an appropriate level of expertise to confirm that it is of an acceptable scientific standard.

# Peer review: Reviewer response for version 2

**DOI:** 10.21956/openreseurope.16588.r30415

**Published:** 2022-11-24

**Authors:** David Queller

**Affiliations:** 1 Department of Biology, Washington University in St. Louis, St. Louis, MO, USA

Nice paper. I am happy with all of the responses except for the following one.

**Comment**. Some of the experiments seemed to have a nested structure that is not reflected in the nested statistical tests. For example, Figure 4 has “two experiments with duplicate plates for each cell line”. Did you just average the two duplicate plates into one data point for the analysis?

**Response:** We used all individual measurements for calculating the averages.

**New Comment: **The individual measurements could be misleading if differences among plates make the measurements within a plate non-independent. To make the case extreme, suppose there is no real treatment effect, but you had some extraneous environmental factor that differed across plates, and you happened to have had high values of this factor for each of the plates in one treatment and low values in the two plates of the other treatments. If this environmental factor affects your result variable, the points in treatment 1 will then be different from those in treatment 2, but this would be because of the environmental differences, with an effective sample size of 2 for each treatment. If you don’t include plate as a nested factor in your statistical analysis you would falsely conclude that there is a difference between treatments. A nested analysis would separate out any effect of plate from treatment so you could see the effect of each. An alternative but less rigorous approach is the examine your pate means within treatments. If these are very similar, plate effects are unlikely to be a problem.

Is the study design appropriate and does the work have academic merit?

Yes

Is the work clearly and accurately presented and does it cite the current literature?

Yes

If applicable, is the statistical analysis and its interpretation appropriate?

Partly

Are all the source data underlying the results available to ensure full reproducibility?

Partly

Are the conclusions drawn adequately supported by the results?

Yes

Are sufficient details of methods and analysis provided to allow replication by others?

Yes

Reviewer Expertise:

Evolutionary biology, microbiology

I confirm that I have read this submission and believe that I have an appropriate level of expertise to confirm that it is of an acceptable scientific standard, however I have significant reservations, as outlined above.

# Response

**Published:** 2022-11-26

**Authors:** Pauline Schaap

**Affiliations:** University of Dundee, UK

I have to apologize. I looked back at the original data and the cell/plaque counts for the two experimental replicates had already been combined before the statistical test was performed. The problem raised by the reviewer therefore does not apply. However, more generally speaking if we would observe large plate or experiment specific variation, we would try to identify the cause and repeat the experiments.

# Peer review: Reviewer response for version 2

**DOI:** 10.21956/openreseurope.16588.r30413

**Published:** 2022-11-24

**Authors:** Thomas Winckler

**Affiliations:** 1 Institute of Pharmacy, Pharmaceutical Biology, University of Jena, Jena, Germany

No comments.

Is the study design appropriate and does the work have academic merit?

Yes

Is the work clearly and accurately presented and does it cite the current literature?

Yes

If applicable, is the statistical analysis and its interpretation appropriate?

Yes

Are all the source data underlying the results available to ensure full reproducibility?

Yes

Are the conclusions drawn adequately supported by the results?

Partly

Are sufficient details of methods and analysis provided to allow replication by others?

Yes

Reviewer Expertise:

Intercellular communication during early development in social amoebas

I confirm that I have read this submission and believe that I have an appropriate level of expertise to confirm that it is of an acceptable scientific standard.

# Peer review: Reviewer response for version 1

**DOI:** 10.21956/openreseurope.16154.r30014

**Published:** 2022-09-20

**Authors:** David Queller

**Affiliations:** 1 Department of Biology, Washington University in St. Louis, St. Louis, MO, USA

This work follows up research on the evolution of multicellularity in the model organism *Dictyostelium discoideum* with work on one of its relative *Polysphondylium pallidum*. These organisms form a multicellular fruiting body with a sterile stalk supporting reproductive spores, a simple form of soma/germline division. Work on *D. discoideum* suggests that autophagy of stalk cells is required for spore development. This is a hint that stalk cells may be feeding spore cells, which would be a novel function of the stalk cells. *Polysphondylium pallidum* offers an opportunity to test the generality of this finding and to see whether autophagy is also important in another dormant structure, cysts, which are lacking in *D. discoideum.*

 Knockouts of two essential autophagy genes did not prevent encystation, though they did reduce it. These knockouts did eliminate spore production confirming that autophagy is indeed essential for spore development in *D. discoideum*. cAMP-induced prespore gene expression was also lost. Finally, chemical inducement of spores resulted in spores that were smaller and less resistant to detergent. These results provide further evidence consistent with spores gaining nutrition from stalk cells. There are other possible interpretations, but the authors are open about this, so readers should not be led to overinterpret. This work should stimulate further research on the topic, potentially showing that early cell differentiation was as much or more about food transfer as about aiding dispersal of the spores.

 I have a few suggestions and question, none of them very major:

- I wonder if these results are relevant to non-fruiting cheater mutants (Ennis *et. al *2000 ^1^, Kuzdzal-Fick *et al* 2011 ^2^). A mutant that does not produce proper stalk cells would not develop on its own, but could be fed by the stalk cells of other clones if developed in mixtures.
- I am curious if the authors have any thoughts on how and when food transfer takes place. Does food have to pass up the stalk somehow, or does the transfer occur earlier?
- I would not use the abbreviations Ddis and Ppal for the species. Readers will be familiar with the actual names but not these abbreviations.
- Figure 1, the “phylogenies” of atg5 and atg7 are misleading. A much better estimate of the phylogeny is shown below them. Atg5 and Atg7 presumably evolved on that same phylogeny (unless there was horizontal gene transfer, which seems unlikely). The differences from the best phylogeny presumably just reflect errors in estimation because of smaller datasets for single genes. The domain structures of atg5 and atg7 should just be mapped on the best phylogeny (this shouldn’t change any real conclusions though).
- Figures with histograms. In my field, authors are moving away from mean/st.dev histograms when the number of data points is small, showing the actual data points instead.
- On page 9 it says, “Only in the mixture with 20% WT were some small fruiting bodies detected. However, none of their spores were G418 resistant, indicating that neither atg7ˉ nor atg5ˉ had formed spores in the chimeras. Their sporulation defect is therefore cell-autonomous.” How does this square with the interpretation that stalk cells are feeding the spores? Wouldn’t we expect stalk cells of the wildtype to be able to nurture the knockouts (see my point about non-fruiting cheaters above)? If you agree, you might want to back off on the feeding interpretation somewhat, or at least mention this discrepancy in the Discussion. (The Discussion is overall thorough and nuanced however). Perhaps more wildtype cells are needed?
- Figure 4 involves a 1-way ANOVA. How many categories, just RI vs knockout?
- Some of the experiments seemed to have a nested structure that is not reflected in the nested statistical tests. For example Figure 4 has “two experiments with duplicate plates for each cell line”. Did you just average the two duplicate plates into one data point for the analysis?
- I found qPCR data deposited but not other data from the paper. Perhaps I am not looking in the right place?

Is the study design appropriate and does the work have academic merit?

Yes

Is the work clearly and accurately presented and does it cite the current literature?

Yes

If applicable, is the statistical analysis and its interpretation appropriate?

Partly

Are all the source data underlying the results available to ensure full reproducibility?

Partly

Are the conclusions drawn adequately supported by the results?

Yes

Are sufficient details of methods and analysis provided to allow replication by others?

Yes

Reviewer Expertise:

Evolutionary biology, microbiology

I confirm that I have read this submission and believe that I have an appropriate level of expertise to confirm that it is of an acceptable scientific standard, however I have significant reservations, as outlined above.

## References

[1] : Dictyostelium amoebae lacking an F-box protein form spores rather than stalk in chimeras with wild type. *Proc Natl Acad Sci U S A* .2000;97(7) : 10.1073/pnas.97.7.3292 3292-7 10.1073/pnas.97.7.3292 10725352PMC16232

[2] : High relatedness is necessary and sufficient to maintain multicellularity in Dictyostelium. *Science* .2011;334(6062) : 10.1126/science.1213272 1548-51 10.1126/science.1213272 22174251

# Response

**Published:** 2022-11-12

**Authors:** Pauline Schaap

**Affiliations:** University of Dundee, UK

We thank the reviewer for his constructive comments which we addressed below

**Comment:** I wonder if these results are relevant to non-fruiting cheater mutants (Ennis et. al 20001, Kuzdzal-Fick et al 20112). A mutant that does not produce proper stalk cells would not develop on its own, but could be fed by the stalk cells of other clones if developed in mixtures.  

**Response:** It is certainly possible that the non-fruiting cheater mutants fail to form spores on their own because they do not form a stalk. However, we are reluctant to cite this as corroborating evidence of the requirement for nutrient flow from stalk cells, since the molecular mechanism of their defective fruiting body formation is unresolved and could also be the result of defective cell-cell communication.  

**Comment:** I am curious if the authors have any thoughts on how and when food transfer takes place. Does food have to pass up the stalk somehow, or does the transfer occur earlier?  

**Response: **That is at the moment hard to say. In the slug stage, the anterior-like cells, which have prestalk characteristics, are intermixed with prespore cells and are in a good position to transfer nutrients. Later, the maturing stalk, where almost complete autolysis of cellular contents occurs, passes straight through the centre of the spore mass and is therefore equally well positioned. Dictyostelia have many ABC transporter genes which could be involved in nutrient secretion and uptake, but other processes like exocytosis of mature autophagosomes by the prestalk cells and fluid uptake through pinocytosis by the prespore cells may also play a role.  A nifty technique for following nutrient flux would be required to answer these questions.  

**Comment:** I would not use the abbreviations Ddis and Ppal for the species. Readers will be familiar with the actual names but not these abbreviations.  

**Response:** The abbreviations have been explained at their first use and we use them very frequently (34 and 64 times for *Ddis* and *Ppal*, respectively. We prefer to retain them to keep sentences compact.  

**Comment:** Figure 1, the “phylogenies” of atg5 and atg7 are misleading. A much better estimate of the phylogeny is shown below them. Atg5 and Atg7 presumably evolved on that same phylogeny (unless there was horizontal gene transfer, which seems unlikely). The differences from the best phylogeny presumably just reflect errors in estimation because of smaller datasets for single genes. The domain structures of atg5 and atg7 should just be mapped on the best phylogeny (this shouldn’t change any real conclusions though).  

**Response:** We should have explained the purpose of the single gene phylogenies better. These are not intended to highlight relationships between Amoebozoa, but to assess whether the homologs identified by BLAST search are likely to be orthologs of atg5 or atg7 (i.e. form a monophyletic clade that also shares the same domain architecture) and did not undergo recent gene duplications (which would require double knock-outs to be made). The single gene phylogenies may reproduce the multigene species phylogeny shown at the bottom of figure 2, but as stated by the reviewer there is often insufficient phylogenetic signal to achieve this. The single gene phylogenies show that the second best BLAST hits are not monophyletic and have different architectures, so we can be sure to be dealing with single *atg5* and *atg7* genes in *P. pallidum. *We altered the text of the first paragraph of the Results section to clarify the issue.  

**Comment: **Figures with histograms. In my field, authors are moving away from mean/st.dev histograms when the number of data points is small, showing the actual data points instead.  

**Response:** That is indeed preferable when there are not too many variables. However, when dealing with three cell lines, each exposed to different treatments, a visually more simple presentation of coloured bars helps the reader keep better track of the outcome of the experiments. All individual measurements are archived in spreadsheets in "ExtendedData_Du_Schaap_MS" for those interested in such detail.  

**Comment: **On page 9 it says, “Only in the mixture with 20% WT were some small fruiting bodies detected. However, none of their spores were G418 resistant, indicating that neither atg7ˉ nor atg5ˉ had formed spores in the chimeras. Their sporulation defect is therefore cell-autonomous.” How does this square with the interpretation that stalk cells are feeding the spores? Wouldn’t we expect stalk cells of the wildtype to be able to nurture the knockouts (see my point about non-fruiting cheaters above)? If you agree, you might want to back off on the feeding interpretation somewhat, or at least mention this discrepancy in the Discussion. (The Discussion is overall thorough and nuanced however). Perhaps more wildtype cells are needed?  

**Response:** The feature that surprised us most in the autophagy mutants and that triggered the present study is their failure to express prespore genes in response to cAMP. This is a defect in cAMP signal transduction in the prespore cells and therefore cell-autonomous. We do not how autophagy affects signal transduction – one possibility is that autophagy removes an inhibitor of a component of the (mostly inknown) signal transduction pathway. However, this early cell-autonomous effect of autophagy prevents us from assessing whether later in development there is also the non cell-autonomous defect of lack of nutrient flow from the stalk cells, that was inferred from the 8Br-cAMP experiment.               

**Comment:**  Figure 4 involves a 1-way ANOVA. How many categories, just RI vs knockout?  

**Response:** We compared all variables against each other (See the component "Statistics" in the Extended Data, Spreadsheet Fig_4Encystation_data+statistics.xlxs), but only the knockout values compared to either of the random integrant values were significantly different from each other  

**Comment:** Some of the experiments seemed to have a nested structure that is not reflected in the nested statistical tests. For example Figure 4 has “two experiments with duplicate plates for each cell line”. Did you just average the two duplicate plates into one data point for the analysis?  

**Response:** We used all individual measurements for calculating the averages  

**Comment:** I found qPCR data deposited but not other data from the paper. Perhaps I am not looking in the right place?  

**Response:** SupdataRNAseq.xlsx was missing, but the other files were there. However, one needed to get back to the page OSF Registries | ExtendedData_Du_MS | Components to be able to reach them. It was the first time that we used this archive and we realized later that the need to create separate components was not necessary and that all .zip files could be listed on a single page. A new registry was created named ExtendedData_Du_Schaap_MS Now after clicking the link in the manuscript in the paragraph "data availibility", click on Files in the left menu bar and then on "Archive of OSF storage" in the main window of the page that opens. Then a listing of all .zip files appears, which can be downloaded inidividually or all together. We also altered the names of the .zip files to include reference to the figure in the main text to which they apply. We hope the underlying data is now easier to navigate.

# Peer review: Reviewer response for version 1

**DOI:** 10.21956/openreseurope.16154.r30015

**Published:** 2022-09-16

**Authors:** Ricardo Escalante

**Affiliations:** 1 C.S.I.C./U.A.M, Instituto De Investigaciones Biomédicas Alberto Sols, Madrid, Spain

In this paper the role of autophagy in *Polysphondylium pallidum* development has been analyzed by disrupting the autophagy genes atg5 and atg7. The authors provide convincing data that spore, and stalk differentiation, and also encystation are dependent on autophagy to a greater or lesser extent. This study is relevant in the field and provides new insights into the role of autophagy in development and differentiation. However, I believe that the interpretation/discussion of the data and conclusions (even the title) should be refined to accurately reflect the experiments.

 1. In my opinion, the conclusion about the possibility that stalk cells nurses the spores through autophagy is not sufficiently supported by the data and should be nuanced. The authors based this conclusion on the lack of viability of spores generated in isolation versus spores from fruiting bodies. I agree with the logical conclusion that some essential signals/metabolites are missing in isolation and that it is likely that some of these signals may emanate from stalk cells. However, the hypothesis that such stalk signals/metabolites are autophagy-dependent is based on the assumption that autophagy is higher in stalk cells. Even if this is true, that does not necessarily mean that autophagy in prespore cells is not relevant for prespore differentiation. Moreover, this point is not even settled in the literature, as no comparison of "autophagy flux" in prespore and prestalk cells has been performed. Furthermore, the mixing experiments between WT and autophagy-deficient cells strongly suggest that the lack of spore differentiation of autophagy-deficient cells is cell autonomous and, therefore, that autophagy in prespore cells is necessary for proper spore differentiation.

 2. The discussion at some point assumes the nursing hypothesis as a true fact to build an evolutionary hypothesis. In my opinion this part of the discussion may be reconsidered or nuanced.

 3. Autophagy has not been assessed in Atg5, Atg7 *P. pallidum* mutants. I think it would be important to have a confirmation of the lack of autophagy with at least one of the several tools optimized for *Dictyostelium discoideum*.

 4. In the abstract and other places it is stated without qualification that stalk differentiation does not require autophagy, but the fact is that stalk morphology is greatly affected and this may be due to defects in proper differentiation. In my opinion, the role of autophagy in stalk differentiation should not be neglected. The same is true for encystation.

 5. Page 6. Detailed description of the promoter-LacZ constructs should be provided. How were they designed to contain all the regulatory elements for proper expression?

 6. Page 9. About the analysis of spores in in atg7 and Atg5 KO strains. It is stated that "No spores differentiated at all". Please explain how spore differentiation was assessed in this experiment.

 7. Statistical significance of the differences should be included where appropriate (e.g., Figures 4 and 5).

Is the study design appropriate and does the work have academic merit?

Yes

Is the work clearly and accurately presented and does it cite the current literature?

Yes

If applicable, is the statistical analysis and its interpretation appropriate?

Partly

Are all the source data underlying the results available to ensure full reproducibility?

Yes

Are the conclusions drawn adequately supported by the results?

Partly

Are sufficient details of methods and analysis provided to allow replication by others?

Yes

Reviewer Expertise:

Autophagy; Dictyostelium

I confirm that I have read this submission and believe that I have an appropriate level of expertise to confirm that it is of an acceptable scientific standard, however I have significant reservations, as outlined above.

# Response

**Published:** 2022-11-12

**Authors:** Pauline Schaap

**Affiliations:** University of Dundee, UK

We thank the reviewer for his careful reading of the manuscript and insightful comments, which we addressed as follows:

**Comment 1:**. In my opinion, the conclusion about the possibility that stalk cells nurses the spores through autophagy is not sufficiently supported by the data and should be nuanced. The authors based this conclusion on the lack of viability of spores generated in isolation versus spores from fruiting bodies. I agree with the logical conclusion that some essential signals/metabolites are missing in isolation and that it is likely that some of these signals may emanate from stalk cells. However, the hypothesis that such stalk signals/metabolites are autophagy-dependent is based on the assumption that autophagy is higher in stalk cells. Even if this is true, that does not necessarily mean that autophagy in prespore cells is not relevant for prespore differentiation. Moreover, this point is not even settled in the literature, as no comparison of "autophagy flux" in prespore and prestalk cells has been performed. Furthermore, the mixing experiments between WT and autophagy-deficient cells strongly suggest that the lack of spore differentiation of autophagy-deficient cells is cell autonomous and, therefore, that autophagy in prespore cells is necessary for proper spore differentiation.  

**Response:** We agree with the reviewers 1 and 2 that since it was not technically feasible to demonstrate nutrient flow from prestalk/stalk to spore cells, the conclusion that  stalk cells nurse the spores should be moderated, We did so by inserting "likely" before "nursed" in the title. We have however not stated that autophagy is not occurring or not important in the prespore cells, only that it is less evident. This statement is derived from comparison of cross section areas occupied by autophagic vacuoles in EM images of slug prestalk and prespore regions (Schaap, 1983 Roux's archives 192, 86–94), which was 4-5 fold less in prespore than prestalk cells, and by the literature cited in the Discussion section. This was Devine & Loomis, 1985 as one of many papers showing that prestalk but not prespore cells strongly stain with neutral red, which typically accumulates in acidic autolysosomes, while the recent paper by Yamada & Schaap 2021 shows both more total and more acidic vesicles containing the autophagy flux marker RFP-GFP-Atg8 in prestalk than prespore cells (Figure S8) as well as more puncta with the autophagy proteins KnkA and Bcas3 in prestalk over prespore cells (Figure 6A). The spore differentiation defect in the autophagy mutants is cell autonomous because they lack the ability to transduce extracellular cAMP to induce prespore differentiation. Since this early step does not occur it cannot be determined whether the later phases – the synthesis of prespore vesicles and spore maturation are cell autonomous or not.

**Comment 2.** The discussion at some point assumes the nursing hypothesis as a true fact to build an evolutionary hypothesis. In my opinion this part of the discussion may be reconsidered or nuanced.  

**​​​​​​​Response: **We have moderated the nursing hypothesis by altering the sentence: "The reliance of spores on nutrients from prestalk/stalk cells is analogous to the reliance of zygotes on co-aggregated haploid cells and provides insight into the early evolution of somatic cells into "The proposed reliance of spores on nutrients from prestalk/stalk cells is analogous to the reliance of zygotes on co-aggregated haploid cells and may provide insight into the early evolution of somatic cells" providing a more nuanced statement.

**Comment 3.** Autophagy has not been assessed in Atg5, Atg7 *P. pallidum* mutants. I think it would be important to have a confirmation of the lack of autophagy with at least one of the several tools optimized for *Dictyostelium discoideum*.  

**​​​​​​​Response:** We did not consider confirmation of lack of autophagy necessary here for several reasons. Both are well-documented essential autophagy genes and the purpose of knocking them out was not to establish whether they also mediate in autophagy in *P.pallidum* but to compare the knock-out phenotypes with those in *D.discoideum* with respect to loss of cAMP induction of prespore gene expression and spore formation (which was the same) and additional effects on *P. pallidum* encystation. The developmental phenotypes of both mutants are similar to each other and to well documented mutants of several autophagy genes in *D.discoideum*, so we considered further confirmation of their role as autophagy genes to be superfluous.

**Comment 4.** In the abstract and other places it is stated without qualification that stalk differentiation does not require autophagy, but the fact is that stalk morphology is greatly affected and this may be due to defects in proper differentiation. In my opinion, the role of autophagy in stalk differentiation should not be neglected. The same is true for encystation.  

**​​​​​​​Response:** We agree. Cells with the vacuolated walled phenotype of stalk cells still differentiated in the atg5- and atg7- mutants, but the stalks themselves were deformed. We have elaborated more on the stalk phenotype in the Abstract. However, in the Results and Discussion section the stalk phenotype of the *P. pallidum* autophagy mutants was properly described. In a previous study we found the stalks of *D. discoideum* *atg7-*, *atg5-* and *atg9-* mutants to be not so aberrant, but these mutants massively overproduced the stalk-like basal disc cells (Yamada and Schaap, Dev Biol 2019 452:114-126).

**Comment 5**. Page 6. Detailed description of the promoter-LacZ constructs should be provided. How were they designed to contain all the regulatory elements for proper expression?  

**​​​​​​​Response:** More detail about the promoter fragments was provided in Methods just above the description of the LacZ constructs detailing the constructs used for expression of *atg5* and *atg7* from their own promoters. We have now included the exact coordinates of the amplified regions relative to the start coding. For *atg7* 1.5 kb of the 2 kb 5' intergenic region was amplified and for *atg5* 0.6 kb of the 1 kb 5' intergenic region was used. Because the *atg5* and *atg7* expression constructs restored wild-type phenotype to the *atg5-* and *atg7-* mutants, respectively, the amplified promoter segments must have contained the elements required for proper expression of the genes.

**Comment 6. **Page 9. About the analysis of spores in in atg7 and Atg5 KO strains. It is stated that "No spores differentiated at all". Please explain how spore differentiation was assessed in this experiment.  

**Response: **We just did not observe any Calcofluor positive elliptical spores in mature cell masses of the atg5- and atg7- mutants. While wild-type spore heads release spores readily when even slightly mechanically disturbed, the cells inside the atg5- and atg7- structures remained very adhesive making it difficult to resolve them individually. To be able to visually demonstrate the absence of spores we have now dissociated the  atg5- and atg7- structures mechanically to visualize individual cells treated with Calcofluor (new figure 3B). This also allowed us to quantitated total and Calcofluor stained cells over several images, which showed that some cysts but very few spores were formed. The numerical data are cited in the relevant paragraph in Results.  

**Comment 7. **Statistical significance of the differences should be included where appropriate (e.g., Figures 4 and 5).  

 ​​​​​​​ **Response:** For figure 4 all variables were compared with each other (15 comparisons for each panel). Since highlighting significant differences between all comparisons would not render the figure easily comprehensible, we preferred here just to state the relevant significant differences in the legend. Note that the statistical tests for all the data are archived in Open Science Framework: ExtendedData_Du_Schaap_MS. In Figure 5 we have now indicated significant differences between mutants or treatments by asterices.

# Peer review: Reviewer response for version 1

**DOI:** 10.21956/openreseurope.16154.r30013

**Published:** 2022-09-12

**Authors:** Thomas Winckler

**Affiliations:** 1 Institute of Pharmacy, Pharmaceutical Biology, University of Jena, Jena, Germany

The “social amoebas” can go through a facultative multicellular life cycle. After aggregation the amoebas differentiate into at least two cell types and form fruiting bodies, in which the stalk cells support a ball of dormant spores that can germinate under favorable environmental conditions. Because the stalk cells of the fruiting bodies do not survive, it may be assumed that nutrient resources of the multicellular organism would need to be made available primarily to the surviving spore cells. In line with this assumption, Du and Schaap describe in their manuscript that autophagy in (pre)stalk cells is required to produce living spores and conclude that nutrients from stalk cells may somehow be transferred to spores to support their survival. The major experimental approach was to disrupt two autophagy genes, atg5 and atg7, in *Polysphondylium pallidum*. This organism was chosen because it forms spores in multicellular fruiting bodies, but single cells can also form single-cell cysts. Both cyst and spore formation require signals that induce cell wall synthesis. Encystation was somewhat less efficient but not fully prevented by removing atg5 or atg7 in *P. pallidum*. On the other hand, the ability of mutants to form spores after multicellular development was completely lost. This suggests that autophagy is important for spore formation.

 The manuscript is well written. The experimental data are well presented and comprehensible, but I have some comments that may help improve the manuscript:

 The authors describe that “Ppal atg7- and atg5- mutants show defective prespore gene expression”. Yet this conclusion comes from the investigation of only one prespore gene, sp45. Similarly, expression of only one prestalk-specific gene was measured to show that autophagy may not affect prestalk genes. The authors refer to some RNA-seq experiments performed previously, but to me it seems unclear whether the effect in atg7 and atg5 on sp45 expression is specific to sp45 or if more prespore genes are affected by autophagy. Have more prespore and prestalk genes been annotated based on similarity to *D. discoideum* genes and could their expression be determined by RNA-seq of developing P. pallidum cells? Perhaps the authors could clarify that.

 The data showing that autophagy genes atg7 and atg5 are required for cAMP-dependent induction of sp45 are convincing, as well as the data showing that production of living spores by treatment with 8Br-cAMP is more efficient after multicellular development than after induction of single cells. However, the authors do not provide experimental evidence that spores form more efficiently in fruiting bodies because they are “nursed” by stalk cells; i.e., that the spores actually receive nutrients from autophagy within stalk cells. Why do the authors find it less likely that lack of atg7 and atg5 in (pre)stalk cells eliminates a signal usually coming from stalk cells that assists terminal differentiation of spores?

 Abstract: I found the term “multicellular spores“ a bit confusing because each spore is a single cell (although formed by multicellular development).

 Fig. 2: In the phylogenetic trees (panel A) it is unclear what abbreviations such as “Phypo”, “PROFUN” or “ACA” stand for. This should be explained in the figure caption.

Is the study design appropriate and does the work have academic merit?

Yes

Is the work clearly and accurately presented and does it cite the current literature?

Yes

If applicable, is the statistical analysis and its interpretation appropriate?

Yes

Are all the source data underlying the results available to ensure full reproducibility?

Yes

Are the conclusions drawn adequately supported by the results?

Partly

Are sufficient details of methods and analysis provided to allow replication by others?

Yes

Reviewer Expertise:

Intercellular communication during early development in social amoebas

I confirm that I have read this submission and believe that I have an appropriate level of expertise to confirm that it is of an acceptable scientific standard.

# Response

**Published:** 2022-11-12

**Authors:** Pauline Schaap

**Affiliations:** University of Dundee, UK

We thank the reviewer for his constructive comments, which we responded as follows:

**Comment 1** The authors describe that “Ppal atg7- and atg5- mutants show defective prespore gene expression”. Yet this conclusion comes from the investigation of only one prespore gene, sp45. Similarly, expression of only one prestalk-specific gene was measured to show that autophagy may not affect prestalk genes. The authors refer to some RNA-seq experiments performed previously, but to me it seems unclear whether the effect in atg7 and atg5 on sp45 expression is specific to sp45 or if more prespore genes are affected by autophagy. Have more prespore and prestalk genes been annotated based on similarity to D. discoideum genes and could their expression be determined by RNA-seq of developing P. pallidum cells? Perhaps the authors could clarify that.  

**Response:** The previously performed RNA-seq experiments are time courses of *P. pallidum* development and encystation and a single experiment with expression in spores and stalk cells. However, there are no data of genes specifically expressed in prestalk and prespore cells or RNAseq data comparing wild-type cells and autophagy mutants. The spore coat gene sp45 is well-documented to be prespore specific by the Ted Cox lab (Gregg and Cox, 2000 in the reference list). It is a member of a family of spore coat proteins, synthesized in prespore vesicles, which also contains the *D. discoideum* prespore markers CotA, CotB and CotC which are all upregulated by cAMP in unison. The family shows much species-specific gene gain and loss (shown in Gloeckner et al., 2016, Nat. Commun. 7:12085 ,Supplementary data 2, page 5) and there are no clear *P. pallidum* orthologs of CotA-C. We are unsure whether all genes in this family are prespore specific and *sp45* is therefore the only confirmed prespore gene thus far. However, we also use staining with polyclonal spore antibodies to show the lack of prespore vesicles in the atg5- and atg7- mutants, demonstrating that our conclusions are not just based on a single gene but on the most relevant hall mark of prespore cells. Along similar lines, the chosen prestalk gene PPL_04427 is a close homolog to the paradigmatic *D.discoideum* prestalk markers *ecmA* and *ecmB *and was demonstrated to be prestalk specific in promoter-lacZ studies with a similar pattern of expression as *ecmA* and *ecmB*. However, other members of the same family were also expressed in spores (see Schilde et al. EvoDevo 2014, 5:34, so we cannot just use a gene based on homology. It should be noted that until recently almost all work on *D. discoideum* pattern formation also used just a few marker genes.  

**Comment 2** The data showing that autophagy genes *atg7* and *atg5* are required for cAMP-dependent induction of *sp45* are convincing, as well as the data showing that production of living spores by treatment with 8Br-cAMP is more efficient after multicellular development than after induction of single cells. However, the authors do not provide experimental evidence that spores form more efficiently in fruiting bodies because they are “nursed” by stalk cells; i.e., that the spores actually receive nutrients from autophagy within stalk cells. Why do the authors find it less likely that lack of atg7 and atg5 in (pre)stalk cells eliminates a signal usually coming from stalk cells that assists terminal differentiation of spores?  

**Response:** The experiment compared spores formed in fruiting bodies to spores induced as single cells by 8Br-cAMP (not 8Br-cAMP after multicellular development). The conclusion that stalk cells nurse the spores is inferred from the available evidence, since we have no means to directly measure how nutrients flow from stalk to spore cells. Also taken into account the comments of reviewer 3, we therefore changed the title into "Autophagy of the somatic stalk cells **likely** nurses the propagating spores of Dictyostelid social amoebas". We consider it less likely that atg7- or atg5- lack a spore inducing signal for the following reasons. The pivotal process in spore maturation is the exocytosis of prespore vesicles, which contain the first layer and further materials for synthesis of the spore coat (see e.g. Alexander et al., Mol Cell Proteomics 2003 2:1156-63). The signals that induce this exocytosis (SDF2, high osmolarity, discadenine) all act to activate PKA and the use of the PKA activator 8Br-cAMP bypasses the requirement for these signals. It is therefore much more likely that the defects of the individually developing spores are caused by lack of sufficient materials to complete spore wall biosynthesis than lack of a signal.  The atg5- and atg7- cells do not induce prespore differentiation at all, so whether they do not secrete maturation inducing signals is the a moot point. In mutants that do not secrete such signals prespore cells differentiate normally, but do not exocytose the prespore vesicles. We have included a few sentences in the Discussion section to clarify our reasoning.  

**Comment 3** Abstract: I found the term “multicellular spores“ a bit confusing because each spore is a single cell (although formed by multicellular development).  

**Response **We changed "multicellular spores" into "spores formed in fruiting bodies"
